# A modelling approach to hepatic glucose production estimation

**DOI:** 10.1371/journal.pone.0278837

**Published:** 2022-12-21

**Authors:** Simona Panunzi, Andrea De Gaetano

**Affiliations:** 1 Laboratorio di Biomatematica, CNR-IASI, Consiglio Nazionale delle Ricerche, Istituto di Analisi dei Sistemi ed Informatica, Rome, Italy; 2 CNR-IRIB, Consiglio Nazionale delle Ricerche, Istituto per la Ricerca e l’Innovazione Biomedica, Palermo, Italy; University of Milano Bicocca, ITALY

## Abstract

Stable isotopes are currently used to measure glucose fluxes responsible for observed glucose concentrations, providing information on hepatic and peripheral insulin sensitivity. The determination of glucose turnover, along with fasting and postprandial glucose concentrations, is relevant for inferring insulin sensitivity levels. At equilibrium (e.g. during the fasting state) the rate of glucose entering the circulation equals its rate of disappearance from the circulation. If under these conditions tracer is infused at a constant rate and Specific Activity (SA) or Tracer to Tracee (TTR) ratio is computed, the Rate of Appearance (RA) equals the Rate of Disappearance (RD) and equals the ratio between infusion rate and TTR or SA. In the post-prandial situation or during perturbation studies, however, estimation of RA and RD becomes more complex because they are not necessarily equal and, furthermore, may vary over time due to gastric emptying, glucose absorption, appearance of ingested or infused glucose, variations of EGP and glucose disappearance. Up to now, the most commonly used approach to compute RA, RD and EGP has been the single-pool model by Steele. Several authors, however, report pitfalls in the use of this method, such as “paradoxical” increase in EGP immediately after meal ingestion and “negative” rates of EGP. Different attempts have been made to reduce the impact of these errors, but the same problems are still encountered. In the present work a completely different approach is proposed, where cold and labeled [6, 6-2H2] glucose observations are simultaneously fitted and where both RD and EGP are represented by simple but reasonable functions. As an example, this approach is applied to an intra-venous experiment, where cold glucose is infused at variable rates to reproduce a desired glycaemic time-course. The goal of the present work is to show that appropriate, if simple, modelling of the whole infusion procedure together with the underlying physiological system allows robust estimation of EGP with single-tracer administration, without the artefacts produced by the Steele method.

## Introduction

Insulin resistance is the common denominator of many pathological conditions including type 2 diabetes (T2DM), obesity, hypertension, hyperlipidemia, atherosclerosis, non-alcoholic fatty liver disease (NAFLD) and polycystic ovarian syndrome. Drugs that improve insulin sensitivity, such as metformin or thiazolidinediones, improve glycemic control by acting in different ways. For instance, while metformin suppresses Endogenous Glucose Production (EGP), thiazolidinediones mainly increase peripheral glucose uptake [1]. Bariatric surgery (of different types) determines diabetes remission both in the short [2, 3] and in the long term [4–6] through reversal of insulin resistance [7]. However, while Roux-en-Y Gastric Bypass mainly reduces hepatic insulin resistance, Bilio-Pancreatic Diversion increases whole-body insulin sensitivity [8, 9], which is mostly determined by skeletal muscle mass [10]. In this context it is clear that the assessment of the differential mechanisms, whereby insulin sensitivity is improved, greatly benefits from the possibility of estimating Endogenous Glucose Production (EGP).

Stable isotopes are currently used to measure glucose fluxes responsible for observed glucose concentrations, providing information on hepatic and peripheral insulin sensitivity: in particular, hepatic insulin sensitivity can be gauged by measuring EGP. During fasting, EGP provides the necessary glucose to maintain euglycemia: the liver, through glycogenolysis and gluconeogenesis, contributes to about 90% of EGP [11], while kidneys and gut are jointly responsible for the remaining 10%. In the postprandial state the liver helps maintain normal glucose levels by removing glucose from the circulatory stream and storing it into glycogen, while EGP is suppressed. For constant plasma insulin, glucagon and growth hormone concentrations, a doubling of plasma glucose levels was observed to inhibit EGP by 42% and stimulate peripheral glucose uptake by 69% in nondiabetic subjects; in T2DM patients EGP was not suppressed and glucose uptake was stimulated by only 49% [12]. Portal venous hyperglycemia and hyperinsulinemia stimulate hepatic glucose uptake [13] and promote net hepatic glycogen synthesis [14]. Experimental data have indicated that extrahepatic signals may also be important in influencing EGP. Lactate and alanine [15], but also glycerol and free fatty acids [16], are gluconeogenetic precursors: higher concentrations of these substrates determine an increase in EGP. Insulin inhibits gluconeogenesis while glucagon and catecholamines stimulate it [17]. In T2DM hyperglucagonemia contributes to EGP and hyperglycemia [18].

The determination of glucose turnover, along with fasting and postprandial glucose concentrations, is relevant for inferring insulin sensitivity levels. At equilibrium (e.g. during the fasting state) the rate of glucose entering the circulation equals its rate of disappearance from the circulation. If under these conditions tracer is infused at a constant rate and Specific Activity (SA) or Tracer to Tracee (TTR) ratio is computed, the Rate of Appearance (RA) equals the Rate of Disappearance (RD) and equals the ratio between infusion rate and TTR or SA. In the post-prandial situation or during perturbation studies, however, estimation of RA and RD becomes more complex because they are not necessary equal and they may also vary over time due to gastric emptying, glucose absorption, appearance of ingested or infused glucose, variations of EGP and glucose disappearance.

Up to the present day, the most commonly used approach to compute RA, RD and EGP has been the single-pool model by Steele [19–21], which has been used by many investigators [22–25]. However, several authors report pitfalls in the use of this method, such as the “paradoxical” increase in EGP immediately after meal ingestion and, more troubling, “negative” rates of EGP [26, 27].

One may attempt to reduce the impact of these errors by using experimental designs where SA or TTR are kept as constant as possible and where sampling is very frequent [28], but this would limit the range of practicable designs and red would increases experimental costs. Other authors have proposed different approaches: Radziuk et al. advocated the use of a two-compartment model [29], with the introduction of an accessible and a slowly exchanging non-accessible compartment, but the same problems were encountered as with the dual-tracer approach. Cobelli et al. [26] proposed the use of three tracers for the estimation of *RA*_*meal*_, EGP and *RD* after the ingestion of a meal: this method is however very complicated in that it requires, in addition to the use of a third tracer, also that pilot studies are conducted and that the intra-venous tracer infusion rates are varied over time so as to keep both oral and endogenous TTR’s constant.

In the present work a completely different, novel model-based approach is proposed, where cold and labeled [6, 6-2H2] glucose observations are simultaneously fitted and where both *RD* as well as EGP are represented by simple but reasonable functions. As an example, this approach is applied to an intra-venous experiment, where cold glucose is infused at variable rates to reproduce a desired glycemic time-course. The goal of the present work is to show that appropriate, if simple, modelling of the whole infusion procedure together with the underlying physiological system allows for a robust estimation of EGP with a single-tracer administration (as in the Steele approach), without the additional complications of double or triple tracer administration and without the artifacts produced by the Steele method itself.

## Materials and methods

### Study sample

Two NGT, two IGT and two T2DM subjects (average BMI of 53.5 *kg*/*m*^2^) participated in an isoglycemic experiment according to a protocol (Trial registration: ClinicalTrials.gov NCT03223129) approved by the Ethical Commitee of the Catholic University School of Medicine Policlinico Gemelli, Rome, Italy. All participants provided written informed consent. Anthropometric characteristics of the six subjects are reported in Table 1. Details of the experiment and procedures are reported in [30]. Briefly, the experiment consisted in administering patients an oral and an intra-venous glucose test. All subjects were studied in the post-absorptive state after a 12-h overnight fast. To collect arterialized venous blood, a retrograde catheter was inserted in a dorsal hand vein, with the hand kept in a warming blanket. A forearm vein of the controlateral arm was catheterized for the infusions. On day 1, At 08,00 h, [6, 6-2H2]glucose was infused as a priming dose of 22 *μmol*/*kg* and then as continuous infusion throughout the whole procedure at 0.22 *μmol*/*kg*/*min*. After 2.5 h of isotope infusion (basal period), oral glucose loads were given and consumed over 5 minutes at intervals of two hours, three times. The three oral loads consisted respectively of 25 g, 75 g and 100 g of glucose in aqueous solution. Glucose concentrations were evaluated before the start of the first oral glucose load and every ten minutes thereafter. On day 2, the procedure started again, similarly to the first day, with [6, 6-2H2] glucose infusion, first with a priming dose of 22 *μmol*/*kg* and then as a continuous infusion throughout the whole procedure at 0.22 *μmol*/*kg*/*min*. After 2.5 h of [6, 6-2H2] glucose continuous infusion (basal period), a 20%*wt*/*vol* adjustable glucose solution was infused at varying rates to match the plasma glucose concentrations obtained during the oral glucose tolerance test performed on the previous day. The 20% dextrose that was infused during the intra-venous glucose infusion test was enriched with 2, 5% of [6, 6-2H2]glucose to minimize changes in glucose isotopic enrichment. During this experimental IV procedure plasma glucose was measured before the start of the adjustable glucose infusion and every five minutes thereafter in order to change the glucose infusion rate so as to obtain the “isoglycemic” pattern. Plasma glucose concentrations were determined by the glucose oxidase method.

**Table 1:** Anthropometric characteristics of the studied subjects.

Subjects	Gender [*M*/*F*]	Age [*years*]	Weight [*kg*]	BMI [*kg*/*m*^2^]	Study Group
1	M	44	160	55.4	NGT
2	F	60	128	48.8	NGT
3	M	55	131	48.1	T2DM
4	M	19	173	53.4	IGT
5	M	36	201	65.4	T2DM
6	M	24	160	49.9	IGT

### Modelling

**Fig 1:**
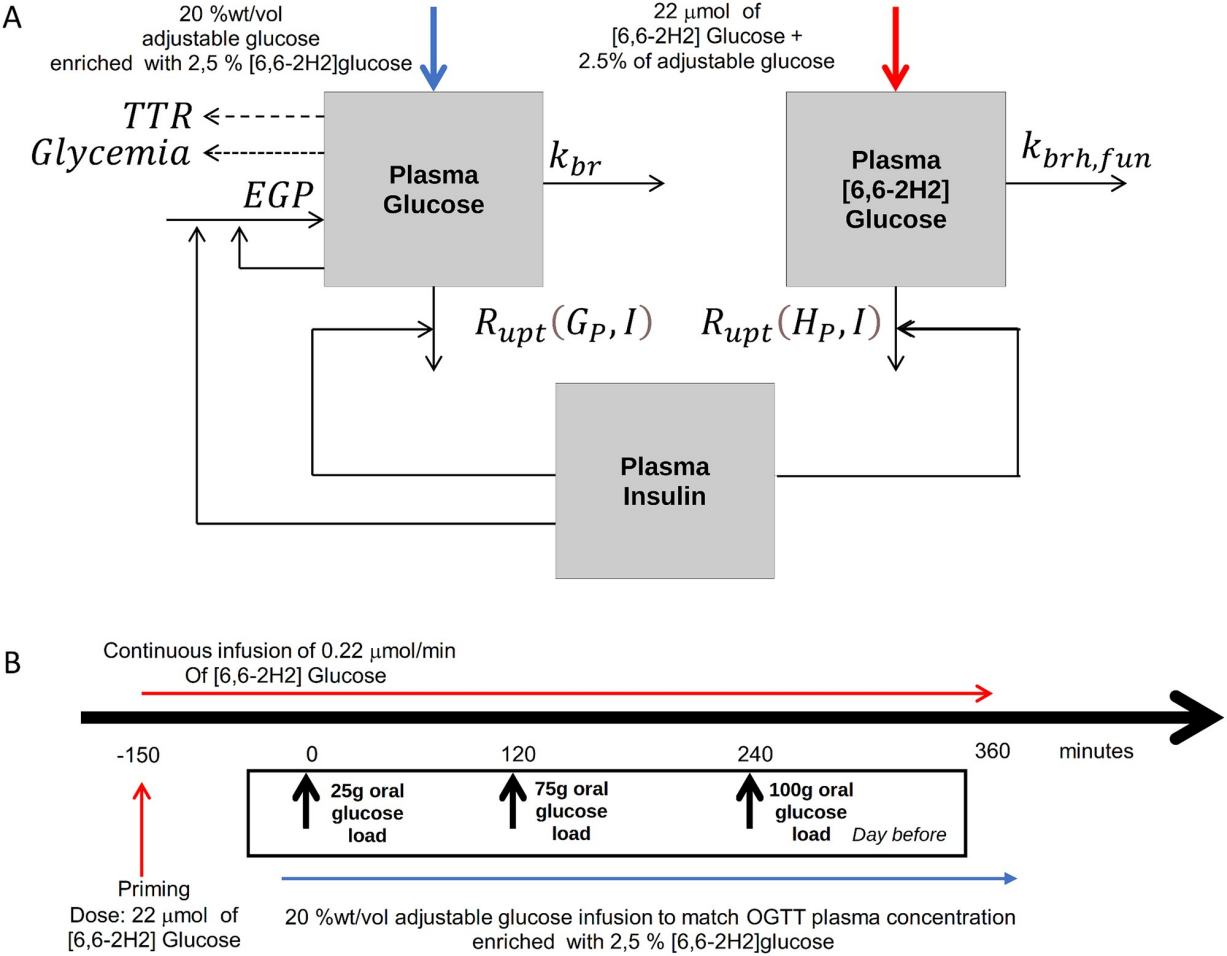
Block diagram. Schematic representation of both the one-compartment glucose model (panel A) and the whole experimental procedure (panel B). The schematic representation of the model summarizes the intravenous procedure performed on day 2.

The objective of the present work was that of developing a model-based approach to estimate glucose liver production during the intravenous experiment described above or similar ones. According to the experimental procedure described in the previous paragraph, the data analysed in the present work is that collected on day 2 when subjects underwent the intra-venous glucose infusion reproducing the glycaemic Oral Glucose Tolerance Test (OGTT) patterns derived from the administration of the three oral glucose loads on day 1 (see Fig 1). The proposed modelling approach, therefore, is applied to the only intra-venous infusion procedure which, from a modelling point of view, is the simplest part of the whole experiment. References to OGTTs are made, therefore, to highlight the three different trends (mirroring the three OGTTs) over time. We note that the proposed modelling approach is independent from the pattern of the infusion, and that, independently from the OGTTs performed on the previous day, it aims at modelling an intra-venous glucose experiment, by implementing the intra-venous inputs, and the consequent physiological response.

We start by considering that in humans water represents about 65% of total body weight (BW) and approximately 5% of BW is water in plasma, 20% in interstitial fluid and 40% in intra-cellular fluid. For a 70 kg man we can therefore state that about 3.5 *ℓ* plus 14 *ℓ* represent the distribution volume for glucose, about 0.25 *ℓ*/*kg* overall. In obese subjects the distribution volume per kilogram body weight is generally smaller.

An average consumption of glucose by the brain of about 104 *g*/*day* is considered [31, 32]. This means about 0.40 *mmol*/*min* (104/1440 × 1000/180 *mmol*/*min*). Upon administration of tracer, the total glucose uptake (sum of ‘cold’ and ‘hot’ glucose uptake, where with ‘hot’ we indicate the [6, 6-2H2]glucose, and with ‘cold’ we indicate the tracee) by the brain can be divided into two components. The hot component, *k*_*brh*,*fun*_/*V*_*gp*_ in equations (4) is the proportion TTR/(1+TTR), where TTR represents the tracer/tracee ratio:

$$\begin{array}{c} k_{brh,fun}\left(t\right)/V_{gp}=(104/1440\times 1000/180)\times TTR(t)/(1+TTR(t)) \end{array}$$

which varies over time as function of TTR(t), while the total uptake in Eq (1), represented by the term *k*_*br*_/*V*_*gp*_ is constant with *k*_*br*_ equal to 0.40 *mmol*/*min* (being *V*_*gp*_ the plasma glucose distribution volume expressed in *ℓ*/*kg*).

Two different models were evaluated: the simplest model includes only one physical glucose compartment, while a more detailed model includes both plasma and interstitial compartments. When evaluating the last model two volumes were thus separately considered: plasma volume, whose typical value is approximately 0.05 *ℓ*/*kgBW*, and interstitial volume, whose typical value is about 0.20 *ℓ*/*kgBW*.

All the model state variables and model parameters are described in Tables 2 and 3, respectively, along with their corresponding units of measurement.

**Table 2:** Model variables and input variables (external inputs) along with their unit of measurement and description.

**Model Variable**	**Units**	**Description**
*G*_*P*_	mM	‘Total’ glucose concentration in plasma
*G*_*I*_	mM	‘Total’ glucose concentration in interstitium
*H*_*P*_	mM	Concentration of infused ‘hot’ glucose [6, 6−^2^ *H*_2_] in plasma
*H*_*I*_	mM	Concentration of infused ‘hot’ glucose [6, 6−^2^ *H*_2_] in interstitium
*Y*	#	Tracer on tracee ratio
*R*_*a*,*gut*_	mmol/kg/min	Glucose absorption from gut
*R*_*a*,*liv*_	mmol/kg/min	Rate of liver glucose production
*R*_*upt*_(*G*_*p*_, *I*)	mM/min	Insulin dependent ‘total’ glucose elimination rate
*R*_*upt*_(*H*_*p*_, *I*)	mM/min	Insulin dependent ‘hot’ glucose elimination rate
*R*_*kid*_	mM/min	Glucose renal extraction
**Input Variable**	**Units**	**Description**
*I*	pM	Insulin plasma concentration (interpolated)
*R*_*inf*_	mmol/kg/min	Infusion of ‘cold’ glucose
*R*_*inf*,*H*_	mmol/kg/min	Infusion of ‘hot’ glucose

**Table 3:** Model parameters along with their unit of measurement, description and typical value.

Parameter	Units	Meaning	Value	Type	Model
*t*_0_	min	Starting time for numerical integration	-150	-	-
*V*_*gp*_	L/kg	Glucose distribution volume for plasma compartment	0.25	free	both
*V*_*gi*_	L/kg	Glucose distribution volume for interstitium compartment	0.14	free	two-comp
*k*_*xGI*,1_	1/min/pM	Insulin dependent tissue Glucose Uptake rate during the first OGTT	0.0001	free	both
*k*_*xGI*,2_	1/min/pM	Insulin dependent tissue Glucose Uptake rate during the second OGTT	0.0001	free	both
*k*_*xGI*,3_	1/min/pM	Insulin dependent tissue Glucose Uptake rate during the third OGTT	0.0001	free	both
*k*_*IG*,*max*_	mmol/kg/min	Maximal Endogenous Glucose Production	0.01	determined (Eqs (7) or (9))	both
λ_*GI*_	1/*mM*/*pM*	Exponential decay constant of Endogenous Glucose Production with increasing in Glycemia and Insulinemia	0.0001	free	one-comp
*K*_*G*_	mM	Glucose value at which the glucose multiplicative effect is the half	6	free	two-comp
*γ*_*G*_	#	Hill exponent, with which the glucose effect decrease with the increase of glucose concenteation	0.01	free	two-comp
*K*_*I*_	pM	Insulin value at which the insulin multiplicative effect is the half	70	free	two-comp
*γ*_*I*_	#	Hill exponent, with which the insulin effect decrease with the increase of insulin concenteation	0.001	free	two-comp
*G*_*Pb*_	mM	Basal glucose plasma concentration	5	free	both
*G*_*Ib*_	mM	Basal glucose interstitium concentration	5	computed (3)	two-comp
*I*_*b*_	pM	Basal insulim concentration	35	observed	both
*k*_*ig*_	1/min	first order transfer rate from plasma to interstitium glucose compartment	0.1	free	two-comp
*k*_*gi*_	1/min	first order transfer rate from interstitium to plasma glucose compartment	0.1	determined (Eq (4))	two-comp
*R*_*inf*0_	mmol/kg/min	‘Cold’ glucose infusion at the starting point	0	from the experimental procedure	both
*Weight*	kg	Body weight	128	observed	both
*k*_*b*_	mmol/kg/min	Rate of uptake of ‘total’ glucose by brain	0.0025	computed	both
*Y*_*b*_	#	Predicted tracer to tracee ratio at the starting of the experimental procedure	0.015	computed (Eq 6)	both
*k*_*bh*,*funb*_	mmol/kg/min	Basal rate of uptake of ‘hot’ glucose by brain	4.5e-05	computed (Eq (1) for TTR(t)=*Y*_*b*_)	both
*R*_*a*,*livb*_	mmol/kg/min	baseline value of *R*_*a*,*liv*_ at initial time (*t*_0_)	0.0053	computed	both
*H*_*Pb*_	mM	Plasma [6, 6−^2^ *H*_2_] glucose concentration at the starting point	0.087	computed	both
*H*_*Ib*_	mM	Interstitial [6, 6−^2^ *H*_2_] glucose concentration at the starting point	0	-	two-comp
*D*_*hot*_	mmol/kg	Infused priming dose of [6, 6−^2^ *H*_2_] glucose at the start of the experimental procedure	0.022	from the experimental procedure	two-comp
*R*_*uptb*_	mM/min	Basal insulin-dependent glucose elimination rate	0.01	computed	both

At time -150 *min*, corresponding to the time of the priming infusion, the system is supposed to be at steady state and all the starting conditions of the model equations are referred to that time point. Some model parameters are determined from the model equations, when the system is in the left neighbourhood of time −150: *t* ∈ (−150−*ϵ*, −150), (*t* = −150^−^).

#### ‘Total’ glucose concentration in plasma

The variation of ‘total’ glucose over time (‘hot’ plus ‘cold’ glucose and indicated with *G*_*P*_(*t*) in the equation below) is determined by the external glucose infusion (*R*_*inf*_), by the insulin-dependent uptake of glucose by tissues (*R*_*upt*_, function of *G*_*P*_(*t*) and of the plasma insulin concentration *I*(*t*)), by the insulin-independent brain uptake (*k*_*br*_) and by the term related to the exchanges between plasma and interstitium when two glucose compartments are considered (with *G*_*I*_(*t*) the total glucose concentration in the interstitium compartment). Because we consider experiments where very high glucose concentrations are not reached, we have set renal glucose extraction (represented in the model with the term *R*_*kid*_) to zero. While in principle this choice could be simplistic, plasma glucose concentration in fact never exceeded the “threshold” of 11 *mM* [33] during the analyzed experiments. Moreover, because in a more general representation of the glucose dynamic the rate of appearance from the gastrointestinal tract could also be considered, this has been introduced in the formalization and indicated with *R*_*a*,*gut*_, set to zero here because no oral administration occurs.

In the presence of additional glucose input from the gastrointestinal tract, or as part of modeling a simple OGTT experiment, *R*_*a*,*gut*_ can be modeled as *f*⋅*G*_*gut*_(*t*)/*V*_*g*_ *p*. Here *f* is the fraction of the amount of glucose administered that is effectively absorbed through the intestine, and *G*_*gut*_(*t*) is the glucose that goes through the intestinal tract, possibly approximated as a series of consecutive compartments representing the length of the entire intestine, starting from the proximal down to the most distal part. Endogenous glucose production is represented by the term *R*_*a*,*liv*_ in Eq (1). The expression for ‘total’ glucose variation is therefore the following:

$$\begin{array}{ccc} \frac{dG_{P}\left(t\right)}{dt} & = & \frac{R_{a,gut}\left(t\right)}{V_{gp}}+\frac{R_{a,liv}\left(t\right)}{V_{gp}}+\frac{R_{inf}\left(t\right)}{V_{gp}}-R_{upt}\left(G_{P}\left(t\right),I\left(t\right)\right)\\ & - & R_{kid}\left(t\right)-\frac{k_{br}}{V_{gp}}-k_{ig}G_{P}\left(t\right)+k_{gi}\frac{V_{gi}}{V_{gp}}G_{I}\left(t\right),\;\;\;\;\;G_{P}\left(-150\right)=G_{Pb} \end{array}$$ (1)

where *R*_*upt*_ [mM] is a piecewise function:

$$\begin{array}{c} R_{upt}\left(G_{P}\left(t\right),I\left(t\right)\right)=\{\begin{array}{ccc} k_{xGI,1}\times G_{P}\left(t\right)\times I\left(t\right) & if & t<120\\ k_{xGI,2}\times G_{P}\left(t\right)\times I\left(t\right) & if & 120\leq t<240\\ k_{xGI,3}\times G_{P}\left(t\right)\times I\left(t\right) & if & t\geq 240 \end{array} \end{array}$$ (2)

with

$$\begin{array}{c} R_{upt}\left(G_{P}\left(-150\right),I\left(-150\right)\right)=R_{uptb}k_{xGI,1}\times G_{P}\left(-150\right)\times I\left(-150\right). \end{array}$$

The function in Eq (2) depends upon three different constant insulin-dependent glucose elimination rates *k*_*xGI*,*j*_ (j = 1,2,3), one for each of the three experimental sub-periods. We hypothesize, therefore, that the insulin sensitivity of a subject may vary over the course of the whole procedure: glucose administration, causing insulin release, would thus have different effects when given repeatedly. While some hypoglycemia-preventing mechanism, limiting over time the effect of circulating insulin, may be associated with oral glucose administration, the actual mechanisms involved in the variation of insulin sensitivity are not the object of the present work, but deserve to be investigated.

The terms −*k*_*ig*_ *G*_*P*_(*t*) and $k_{gi}\frac{V_{gi}}{V_{gp}}G_{I}\left(t\right)$ in Eq (1) represent the exchanges between the plasma and the interstitium compartment, with parameter *k*_*ig*_ indicating the rate of transfer from the plasma to the interstitium and parameter *k*_*gi*_ the constant transfer rate from the interstitium to plasma.

Finally, in the one-compartment glucose model, since there is no interstitial compartment, the last two terms in Eq (1) must be eliminated.

#### ‘Total’ glucose concentration in the interstitium

Different mathematical models for the description of the equilibrium between plasma glucose versus interstitial glucose have been proposed in literature. These models are based on the assumption of free diffusion of glucose molecules between plasma and interstitium, and of glucose uptake from the interstitial fluid by tissue cells [34–37]. Rebrin and Steil proposed a “two-compartment” formulation, where interstitium and plasma are two independent compartments separated by a diffusional barrier which glucose is free to traverse, based on its concentration gradient [36, 38, 39]. In this formulation glucose is cleared from the interstitial fluid by tissue cells, at a rate dependent on the interstitial glucose concentration itself. We adopted essentially the same formulation, assuming no external loss from the interstitium. Moreover we hypothesized that the two compartments are at equilibrium at steady state so that their concentrations before the beginning of the experimental procedure are the same. Transfer of glucose from plasma to interstitium and vice versa is described by the transfer rate constants *k*_*ig*_ and *k*_*gi*_.

$$\begin{array}{c} \frac{dG_{I}\left(t\right)}{dt}=k_{ig}\frac{V_{gp}}{V_{gi}}G_{P}\left(t\right)-k_{gi}G_{I}\left(t\right),\;\;\;\;\;G_{I}\left(-150\right)=G_{Ib}=G_{Pb} \end{array}$$ (3)

From equilibrium conditions on Eqs (1) and (3) it follows that two parameters can be determined:

$$\begin{array}{c} k_{gi}=\frac{k_{ig}G_{Pb}V_{gp}}{V_{gi}G_{Ib}}=k_{ig}\frac{V_{gp}}{V_{gi}} \end{array}$$

and

$$\begin{array}{c} R_{a,livb}=R_{upt}\left(G_{Pb},I_{b}\right)V_{gp}+k_{b} \end{array}$$

where *R*_*ab*,*liv*_ is the basal endogenous glucose production. The last equation is also valid for the one-compartment glucose model.

#### Plasma concentration of infused ‘hot’ [6, 6−^2^ *H*_2_] glucose

The isotopic hypothesis is that ‘hot’ [6, 6−^2^ *H*_2_] glucose has the same metabolic destiny as ‘cold’ glucose, so that, apart from the terms related to the Endogenous Glucose Production and forcing input functions, the variations of ‘hot’ glucose can be described, in analogy with Eq (1), by the following equation:

$$\begin{array}{ccc} \frac{dH_{P}\left(t\right)}{dt} & = & \frac{R_{inf,H}\left(t\right)}{V_{gp}}-R_{upt}\left(H_{P}\left(t\right),I\left(t\right)\right)+0.025\times \frac{R_{inf}\left(t\right)}{V_{gp}}-\frac{k_{brh,fun}\left(t\right)}{V_{gp}}+\\ & - & k_{ig}H_{P}\left(t\right)+k_{gi}\frac{V_{gi}}{V_{gp}}H_{I}\left(t\right),\;\;\;\;\;H_{P}\left(-150\right)=H_{Pb}=\frac{D_{hot}}{V_{gp}} \end{array}$$ (4)

where

$$\begin{array}{c} D_{hot}=22\times 10^{-3}mmol/kg. \end{array}$$ (5)

As described before, a priming dose of 22 *μmol*/*kg* was infused at the start of the experimental procedure (*t* = −150 *min*) and it is represented in the model as the initial condition in Eq (4); the term *R*_*inf*,*H*_ represents instead the continuous infusion of 0.22 *μmol*/*kg*^−1^ administered throughout the whole procedure, as described in the subsection “Study Sample”.

#### Interstitial concentration of infused [6, 6−^2^ *H*_2_] glucose

In the same way as for ‘total’ glucose, a linear transfer between plasma and interstitial compartments is considered for ‘hot’ glucose, with the difference that while ‘total’ glucose is assumed at equilibrium at time *t*_0_, ‘hot’ glucose starts at *t*_0_ from a concentration of zero, introducing a delay with which ‘hot’ interstitial glucose concentrations follow ‘hot’ plasma glucose concentrations:

$$\begin{array}{c} \frac{dH_{I}\left(t\right)}{dt}=k_{ig}\frac{V_{gp}}{V_{gi}}H_{P}\left(t\right)-k_{gi}H_{I}\left(t\right),\;\;\;\;\;H_{I}\left(-150\right)=H_{Ib}=0 \end{array}$$

#### Tracer ([6, 6−^2^ *H*_2_] glucose) to tracee ratio

An external substance, the tracer, is used to follow a natural substrate, the tracee, when such a substance exhibits the identical metabolic fate as the tracee. Tracers used in human research are usually identical to the tracee, except that one or more atoms differ from the natural form. The assumption that tracers, isotopes, are treated in identical fashion as their counterparts is at the basis of tracer methodology. The isotopes fall into two classes: radioactive and stable. When dealing with radioactive isotopes the unit of enrichment is the specific activity, SA:

$$\begin{array}{c} SA=\frac{tracer}{tracee}=\frac{dpm}{\mu mol} \end{array}$$

Since scintillation counting is able to detect small quantities of radioisotope, the infused tracer is essentially “massless” and the amount of tracer is represented by disintegrations per minute (*dpm*) [40]. When dealing with stable isotopes, large amounts of labeled compound are necessary. The analogue of SA when stable isotopes are considered is the Tracer to Tracee Ratio (TTR), that is the ratio of the concentrations of tracer to tracee.

From time 0 onwards, the Tracer to Tracee ratio (TTR) is measured every 20 minutes and in the present modelling is given by the ratio of ‘hot’ glucose over ‘cold’ glucose:

$$\begin{array}{c} Y\left(t\right)=\frac{H_{P}\left(t\right)}{\left(G_{P}\left(t\right)-H_{P}\right)},\;\;\;\;\;Y\left(-150\right)=Y_{b}=H_{P}\left(-150\right)/\left(G_{P}\left(-150\right)-H_{P}\left(-150\right)\right) \end{array}$$

Y represents the model prediction of the observed TTR’s. Because ‘cold’ glucose concentrations were measured every 10 minutes, missing data were estimated by interpolation. From observed TTR’s and observed glucose concentrations, observed ‘hot’ glucose concentrations were derived and used in the parameter estimation procedure. Estimation was performed by minimizing a loss function incorporating deviations between observed ‘total’ glucose concentrations, ‘hot’ glucose concentrations and their respective model predictions, see below.

#### Infusion of ‘cold’ glucose

Infusion of ‘cold’ glucose appears in both Eqs (1) and (4) and is indicated with *R*_*inf*_. It is not properly only ‘cold’ glucose since the 20% dextrose infused was enriched with 0,25% of [6, 6-2H2] glucose. This forcing input function is derived from the data, transformed to match the unit of measurements adopted by the experimenters and those adopted in the present work (i.e. from *ml*/*h* to *mmol*/*kg*/*min*). Considering that the infused solution was at 20%*wt*/*vol*, measurements were multiplied by the factor *M*_*inf*_ = 1000×0.2/(180×*Weight* × 60), where Weight is the subject’s Body Weight in *kg*.

#### Rate of liver glucose production

Two formulations of the Rate of Appearance from the liver (that is Endogenous Glucose Production, EGP) have been tested. Liver glucose production from precursors is mediated by the inhibitory effects of insulin and of glucose itself. Hyperinsulinemia is moreover accompanied by suppression of glycogenolysis. Even if some studies suggest that insulin acts on EGP suppression through an extrahepatic route, such as via the suppression of the liberation of FFA’s from adipose tissue [41, 42], the adopted formulations synthetically model the effect of insulin and glucose directly on EGP.

A first mathematical formalization may adopt *multiplication effects*: EGP decreases from a maximal production rate, indicated with *k*_*IG*,*max*_ ([*mmol*/*kg*/*min*]), towards zero for increasing insulin and glucose concentrations according to the following equation:

$$\begin{array}{c} R_{a,liv}\left(t\right)=k_{IG,max}\frac{K_{G}^{\gamma_{G}}}{G_{P}{\left(t\right)}^{\gamma_{G}}+K_{G}^{\gamma_{G}}}\frac{K_{I}^{\gamma_{I}}}{I{\left(t\right)}^{\gamma_{I}}+K_{I}^{\gamma_{I}}} \end{array}$$ (6)

which, from the steady-state condition derived from Eq (1), leads to the following expression for the parameter *k*_*IG*,*max*_:

$$\begin{array}{c} k_{IG,max}=\frac{\left(V_{gp}\times k_{xGI,1}\times G_{P}\left(-150\right)\times I\left(-150\right)+k_{b}\right)}{\left[\frac{K_{G}^{\gamma_{G}}}{G_{P}{\left(-150\right)}^{\gamma_{G}}+K_{G}^{\gamma_{G}}}\frac{K_{I}^{\gamma_{I}}}{I{\left(-150\right)}^{\gamma_{I}}+K_{I}^{\gamma_{I}}}\right]} \end{array}$$ (7)

Adopting the above formulation requires the estimation of four free parameters related to EGP modeling. With the aim of reducing the number of free parameters to be estimated, so as to reduce identifiability problems due to overparametrization, a simpler formulation, requiring only two parameters, has been evaluated and then adopted as the definitive formulation:

$$\begin{array}{c} R_{a,liv}\left(t\right)=k_{IG,max}e^{-\lambda_{GI}\times I\left(t\right)\times G_{P}\left(t\right)} \end{array}$$ (8)

which, from the steady state condition of Eq (1) implies:

$$\begin{array}{c} k_{IG,max}=\frac{\left(V_{gp}\times k_{xGI,1}\times G_{P}\left(-150\right)\times I\left(-150\right)+k_{b}\right)}{e^{\left(-\lambda_{GI}I\left(-150\right)G_{P}\left(-150\right)\right)}} \end{array}$$ (9)

Given that the parameter *k*_*IG*,*max*_ can be thus determined, the single free parameter to be estimated in Eq (8) is λ_*GI*_.

#### Infusion of ‘hot’ glucose

After a priming dose of [6, 6-2H2]glucose, administered at time t=-150, a continuous infusion of ‘hot’ glucose is given during the whole procedure. The forcing function in this modelling formulation has been indicated with *R*_*inf*,*H*_ ([*mmol*/*kg*/*min*]) and appears as the first term in Eq (4), constant throughout the whole procedure. The 0.25% of the forcing function *R*_*inf*_ contributes to the ‘hot’ glucose infusion. The priming dose has been introduced instead in the model as the initial condition of the same equation.

#### Insulin plasma concentration

Insulin concentrations were measured at times 0, 20, 60, 100, 140, 180, 220, 260, 300, 340 minutes. Modelling of insulin secretion would in all likelihood prove beneficial from the point of view of overall model robustness, but a detailed discussion of this aspect would cloud the present issue. For the purpose of this work we have therefore performed a linear interpolation of the observed measurements, smoothing the obtained vector with a Gaussian-weighted moving average filter. The insulin forcing function was denoted with *I*(*t*) ([*pM*]).

### Estimation

Model parameters, their descriptions, units of measurements and corresponding plausible values are reported in Table 3. The last column of the table shows if the parameter is “free” (and hence to be estimated), “determined” from the steady state conditions or “computed” on the basis of initial conditions. When the parameter is “determined”, the corresponding equation in the manuscript is also reported. When it is “computed“, the computation is shown in the table. Fig 1 shows a schematic representation of both the experimental procedure and its modelling approach for the one-compartment glucose model. The model was adapted to data by minimizing the weighted sum of squared deviations of the observed ‘total’ glucose concentrations and ‘hot’ glucose concentrations data from their respective predictions, with weights the inverse of the squared predictions. Estimation was also performed introducing serial correlation within each individual. The hypothesised correlation matrix is:

$$\begin{array}{c} Corr\left(y_{i1},y_{i2}\right)=exp\left(-\alpha_{1}|t_{i,1}-t_{i,2}|\right)=C\left(\alpha \right) \end{array}$$ (10)

where observations *y*_*i*1_ and *y*_*i*2_ on the *i*−*th* subject are taken at times *t*_*i*,1_ and *t*_*i*,2_, respectively. Such a matrix accommodates situations of not equally spaced observations and of a correlation that decreases with increasing lags. If the model for correlation is given by (10) then the covariance for the observation vector *y*_*i*_ is specified by the following expression:

$$\begin{array}{c} Cov\left(y_{i}\right)=\sigma^{2}Diag{\left({\hat{y}}^{2}\left(\theta \right)\right)}^{1/2}C\left(\alpha \right)Diag{\left({\hat{y}}^{2}\left(\theta \right)\right)}^{1/2}=\sigma^{2}\times S^{-1} \end{array}$$ (11)

with *θ* the parameter vector of the model free parameters. Estimation of parameters *σ*, *α* and *θ* was carried out by a recursive procedure which implements subsequent steps:

1. derive an initial estimates $\hat{\theta }$ of *θ* by means of an Ordinary Least Squares or Weighted Least Squares approach;
2. use $\hat{\theta }$ to obtain an estimate for *α* and *σ* with the pseudolikelihood method which minimizes the following function:
  $$\begin{array}{c} PL\left(\hat{\theta },\alpha ,\sigma \right)=log|\sigma^{2}Cov|+{\left(y-f\left(\hat{\theta }\right)\right)}^{T}Cov^{-1}\left(y-f\left(\hat{\theta }\right)\right) \end{array}$$ (12)
3. compute the covariance matrix *Cov* with $\hat{\alpha }$ and $\hat{\sigma }$ from the previous step in (11) and use it to perform a Weighted Least Squares estimation with weights in *Cov*^−1^
4. repeat step 2) and step 3) until convergence.

In any case the final estimation of *σ*^2^ is computed on the estimation of *θ* and *α* according:

$$\begin{array}{c} {\hat{\sigma }}^{2}=\frac{1}{n-p}{\left(y-f\left(\hat{\theta }\right)\right)}^{T}S^{-1}\left(\hat{\theta },\hat{\alpha }\right)\left(y-f\left(\hat{\theta }\right)\right) \end{array}$$ (13)

Under the hypothesis of normally distributed errors, the approximate covariance matrix $\Sigma_{\hat{\theta }}$ of $\hat{\theta }$ is given by:

$$\begin{array}{c} \Sigma_{\hat{\theta }}={\hat{\sigma }}^{2}{\left[J^{T}S^{-1}\left(\hat{\theta },\hat{\alpha }\right)J\right]}^{-1} \end{array}$$ (14)

where *J* is the Jacobian, that is the (*n*×*p*) matrix with *jth* row equal to $f{\left(x_{j},\theta \right)}^{{}^{\prime }}$, with *n* and *p* the number of observations and the number of free parameters, respectively.

Free parameters to be estimated in the final adopted mathematical formulation were *V*_*gp*_, *k*_*xGI*,*j*_, for *j* = 1, 2, 3, *G*_*Pb*_ and λ_*GI*_. When using the two-compartments glucose model, two more parameters had to be estimated: *V*_*gi*_ and *k*_*ig*_. The use of the *multiplication effect* would required the estimation of three additional parameters: four new parameters would appear (*K*_*I*_, *K*_*G*_, *γ*_*I*_ and *γ*_*G*_), while one (λ_*GI*_) would disappear. The remaining parameters were determined by steady state conditions or were set to values either known from the literature (this is the case for the glucose uptake by the brain, see the “Modelling” section above) or derived according to the experimental design (such as some initial conditions).

## Results

The results related to the model including *multiplicative effects* have not been reported: although the fittings obtained were good, parameter estimates exhibited large variations among the studied subjects, and good model performance on the same subject could be obtained with very different values of the four parameters (*K*_*G*_, *K*_*I*_, *γ*_*G*_, *γ*_*I*_), leading to the conclusion that this model was overparametrized. All results detailed below refer therefore to the model without multiplicative effects.

**Fig 2:**
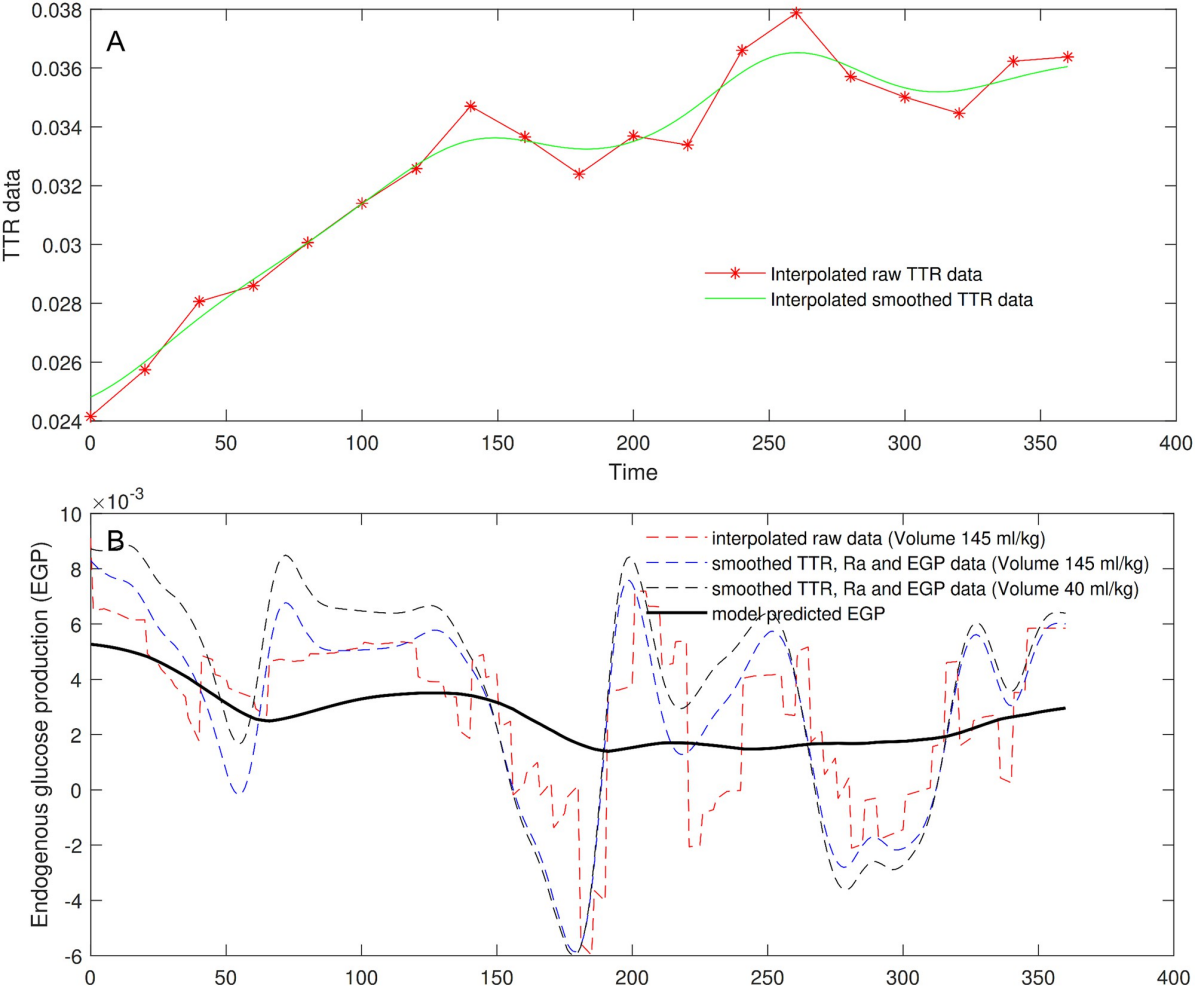
Endogenous glucose production. TTR data (Panel A), only interpolated (dashed red line) and smoothed (continuous green line); EGP computation from the equation of Steele in case TTR data are smoothed and in case they are only interpolated (Panel B). Four different trends of EGP were computed using not smoothed (dashed red line) or smoothed (blue and black dashed lines) TTR data with different glucose distribution volumes (145 ml/kg or 40 ml/kg, respectively), and derived by the model predictions (continuous black line).

Fig 2 shows the EGP computation from the Steele equation, both in the case TTR data are smoothed and in the case they are only interpolated (continuous green line and red dashed line, respectively, in Panel A). The figure reports four different trends of the EGP calculated by also varying the volume of distribution for glucose. According to [43], reasonable values for the volume could range indeed from 40 (plasma volume) to 230 ml/kg (extracellular fluid volume). The value often used is 145 ml/kg, which corresponds to a proportion p = 0.63 of the total volume (i.e., 230 ml/kg). The figure shows the EGP trend obtained with raw data after they have been only interpolated in the entire interval of observation (dashed red line) by using the standard volume of 145 ml; the dashed blue and black lines refer to computations performed by using smoothed interpolated TTR data, as well as smoothed Ra and EGP data, with distribution volumes equal to 145 ml and 40 ml, respectively; the continuous black line represents the predicted EGP estimated with the proposed modelling approach.

**Fig 3:**
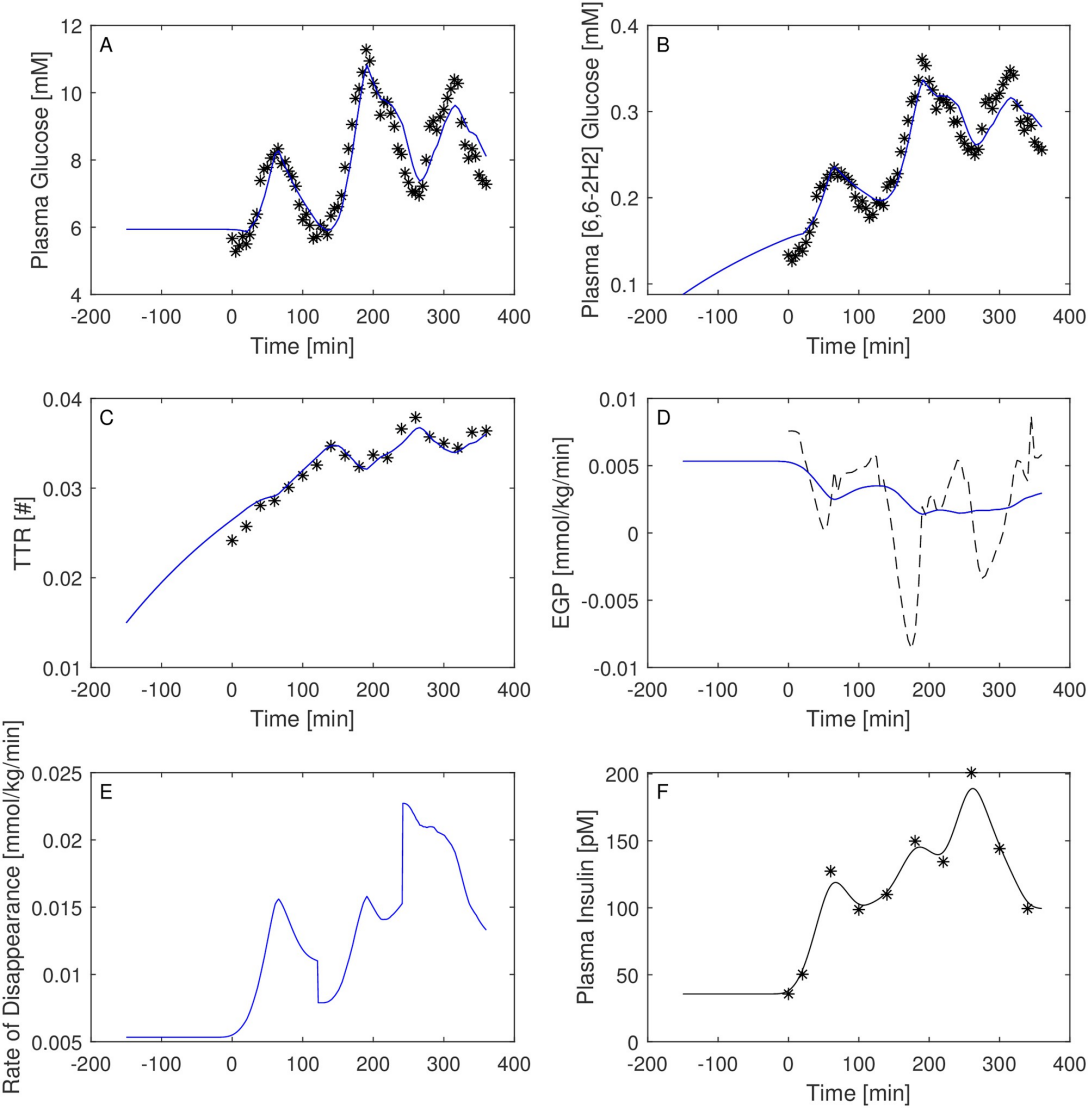
Predictions for Subject 1. Observed (asterisks) and predicted (line) variables over time (NGT subject). EGP: Endogenous Glucose Production; TTR: Tracer to Tracee ratio. Plasma glucose concentration (Panel A); Plasma [6, 6-2H2]glucose (Panel B); TTR (Panel C); EGP (Panel D), dashed line is the predicted EGP with the Steele model; Rate of disappearance (panel E); Plasma Insulin concentrations (Panel F). All the observed points refer to data measured on day 2. Plasma glucose concentrations (panel A) are derived from the glucose intra-venous infusion to match plasma glucose observations from the OGTTs of day 1. Black lines in panels A, B, C, D and E are model predictions from WLS estimation procedure; blue lines derive from the estimation approach which uses autocorrelated errors.

**Fig 4:**
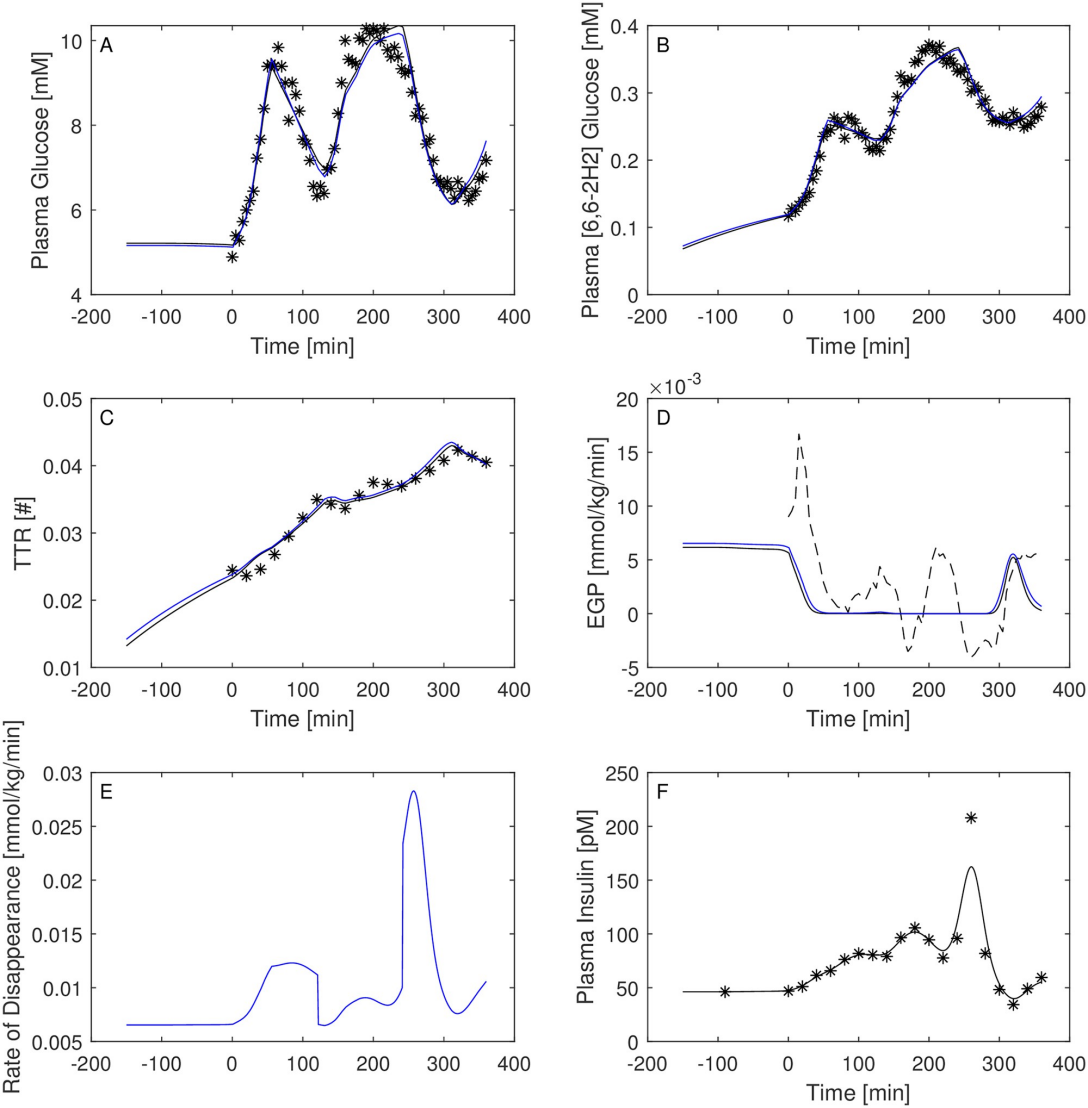
Predictions for Subject 2. Observed (asterisks) and predicted (line) variables over time (NGT subject). EGP: Endogenous Glucose Production; TTR: Tracer to Tracee ratio. Plasma glucose concentration (Panel A); Plasma [6, 6-2H2]glucose (Panel B); TTR (Panel C); EGP (Panel D), dashed line is the predicted EGP with the Steele model; Rate of disappearance (panel E); Plasma Insulin concentrations (Panel F). All the observed points refer to data measured on day 2. Plasma glucose concentrations (panel A) are derived from the glucose intra-venous infusion to match plasma glucose observations from the OGTTs of day 1. Black lines in panels A, B, C, D and E are model predictions from WLS estimation procedure; blue lines derive from the estimation approach which uses autocorrelated errors.

**Fig 5:**
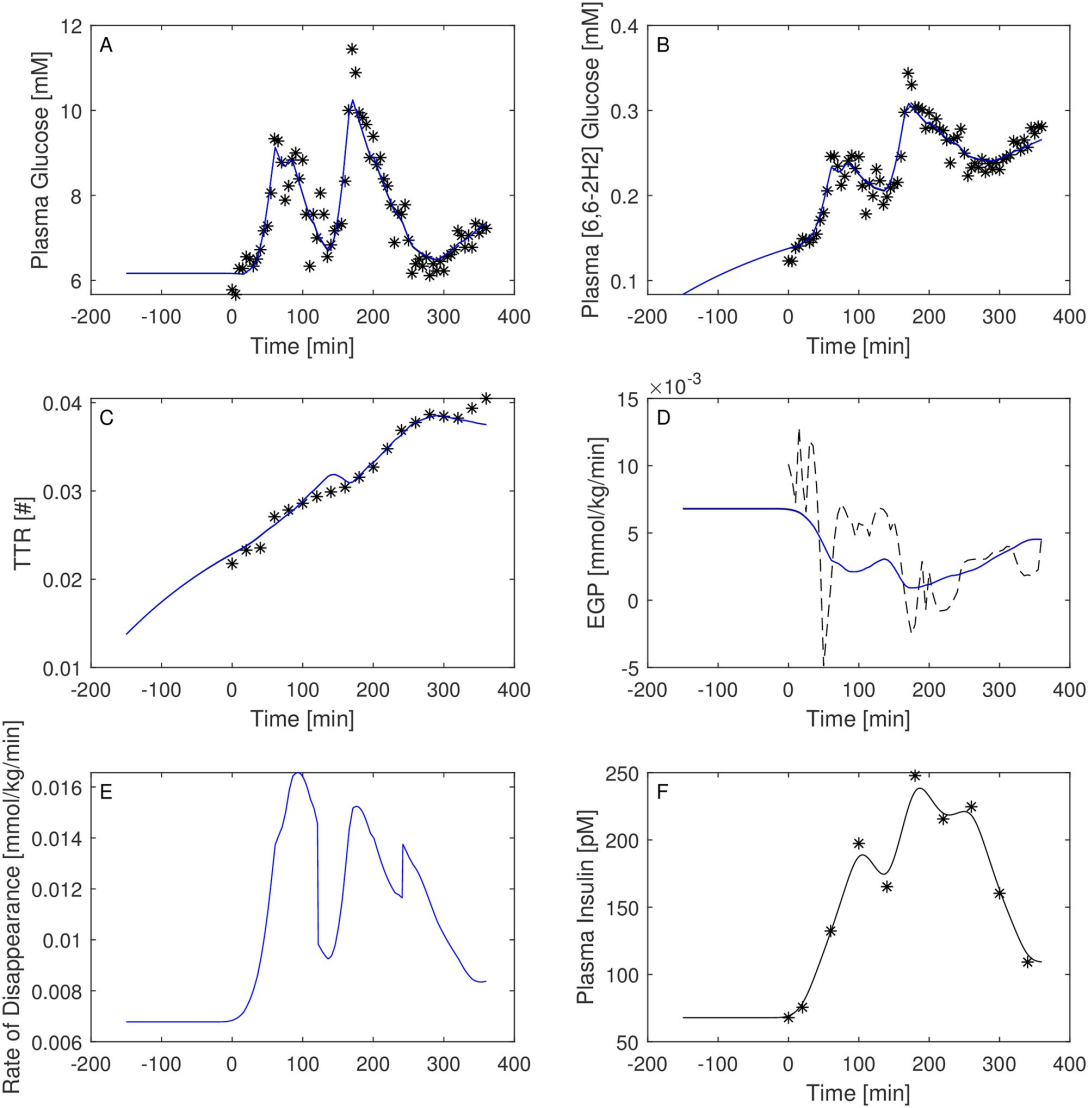
Predictions for Subject 3. Observed (asterisks) and predicted (line) variables over time (T2DM subject). EGP: Endogenous Glucose Production; TTR: Tracer to Tracee ratio. Plasma glucose concentration (Panel A); Plasma [6, 6-2H2]glucose (Panel B); TTR (Panel C); EGP (Panel D), dashed line is the predicted EGP with the Steele model; Rate of disappearance (panel E); Plasma Insulin concentrations (Panel F). All the observed points refer to data measured on day 2. Plasma glucose concentrations (panel A) are derived from the glucose intra-venous infusion to match plasma glucose observations from the OGTTs of day 1. Black lines in panels A, B, C, D and E are model predictions from WLS estimation procedure; blue lines derive from the estimation approach which uses autocorrelated errors

**Fig 6:**
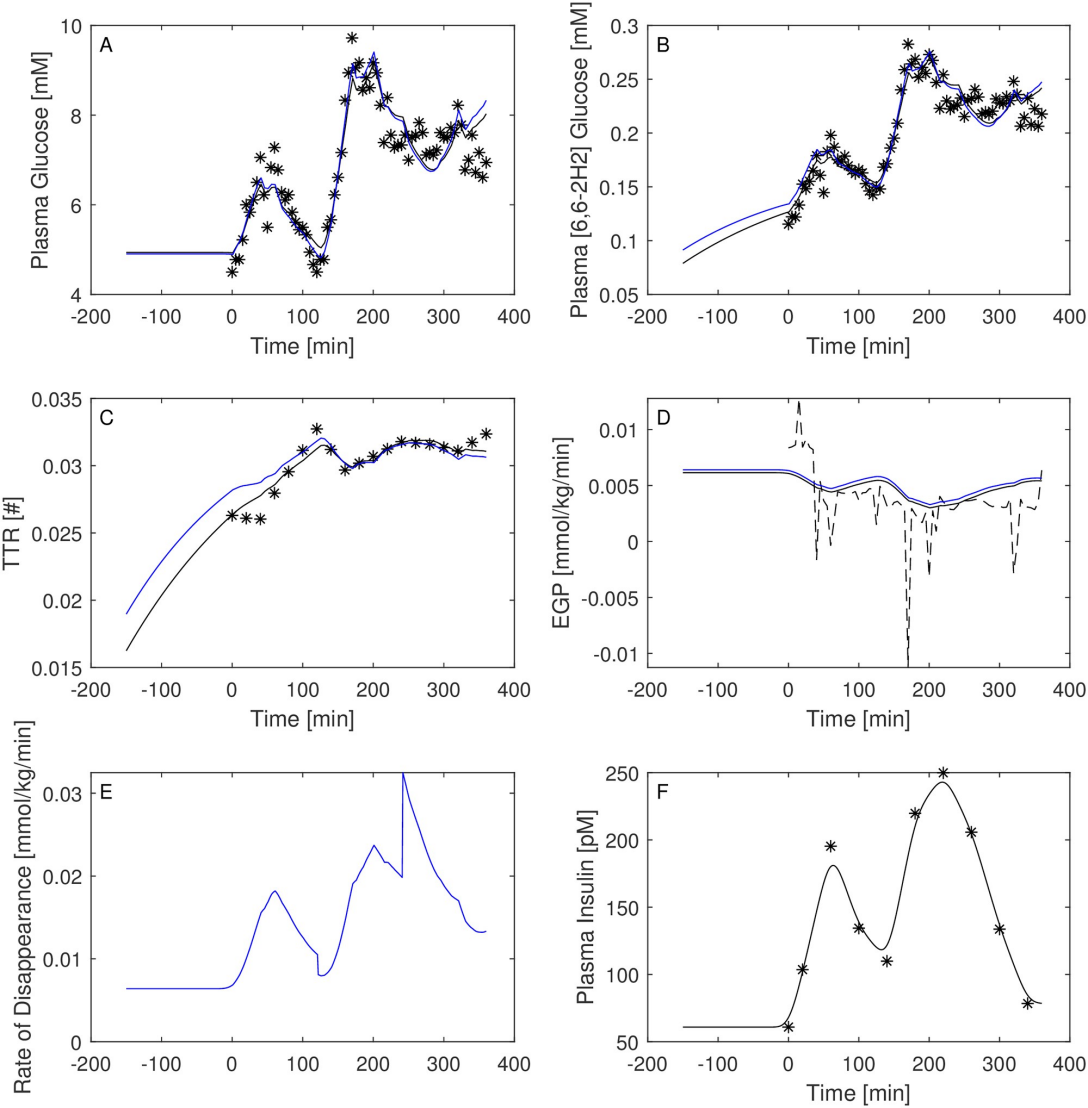
Predictions for Subject 4. Observed (asterisks) and predicted (line) variables over time (IGT subject). EGP: Endogenous Glucose Production; TTR: Tracer to Tracee ratio. Plasma glucose concentration (Panel A); Plasma [6, 6-2H2]glucose (Panel B); TTR (Panel C); EGP (Panel D), dashed line is the predicted EGP with the Steele model; Rate of disappearance (panel E); Plasma Insulin concentrations (Panel F). All the observed points refer to data measured on day 2. Plasma glucose concentrations (panel A) are derived from the glucose intra-venous infusion to match plasma glucose observations from the OGTTs of day 1. Black lines in panels A, B, C, D and E are model predictions from WLS estimation procedure; blue lines derive from the estimation approach which uses autocorrelated errors.

**Fig 7:**
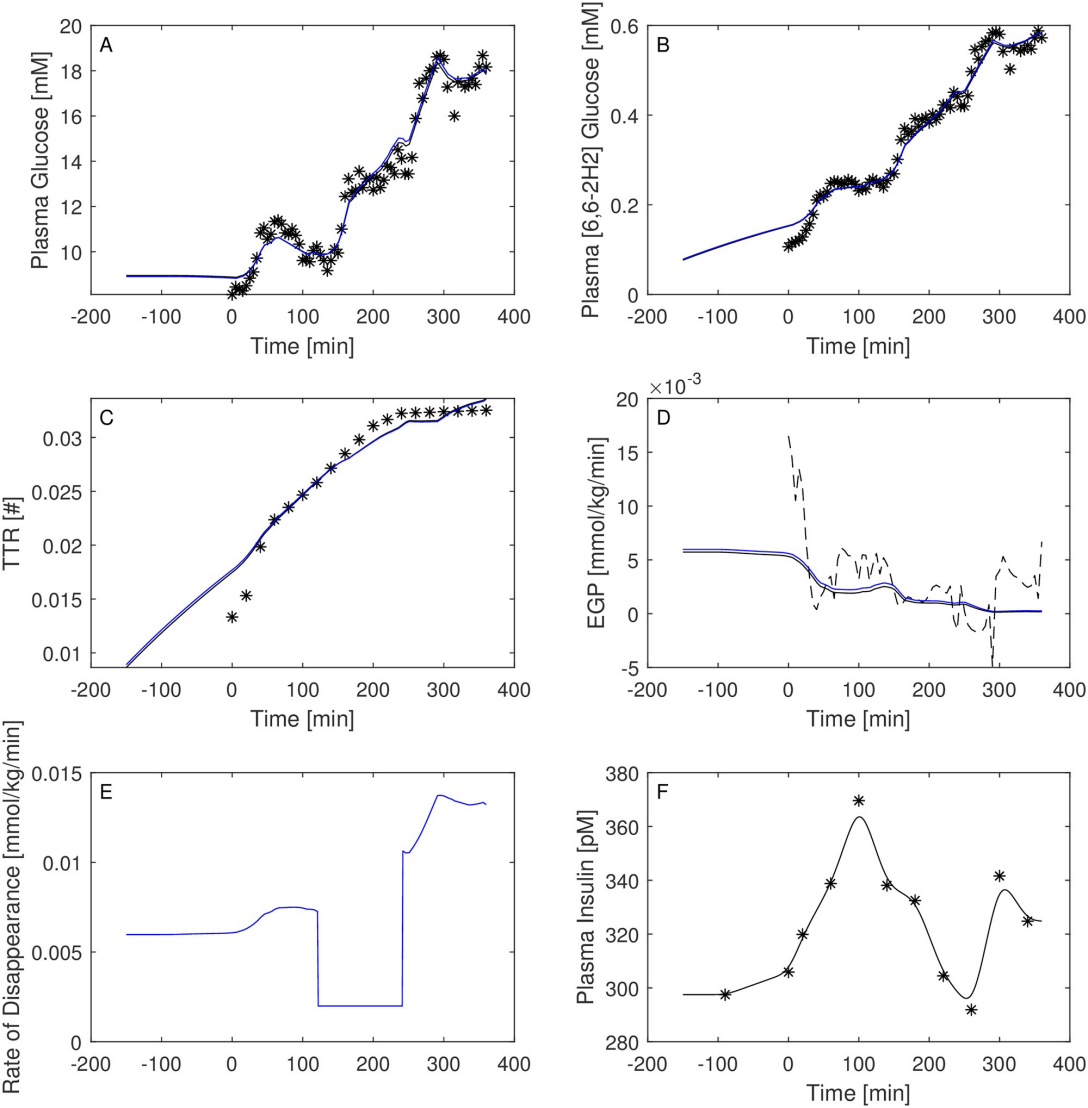
Predictions for Subject 5. Observed (asterisks) and predicted (line) variables over time (T2DM subject). EGP: Endogenous Glucose Production; TTR: Tracer to Tracee ratio. Plasma glucose concentration (Panel A); Plasma [6, 6-2H2]glucose (Panel B); TTR (Panel C); EGP (Panel D), dashed line is the predicted EGP with the Steele model; Rate of disappearance (panel E); Plasma Insulin concentrations (Panel F). All the observed points refer to data measured on day 2. Plasma glucose concentrations (panel A) are derived from the glucose intra-venous infusion to match plasma glucose observations from the OGTTs of day 1. Black lines in panels A, B, C, D and E are model predictions from WLS estimation procedure; blue lines derive from the estimation approach which uses autocorrelated errors.

**Fig 8:**
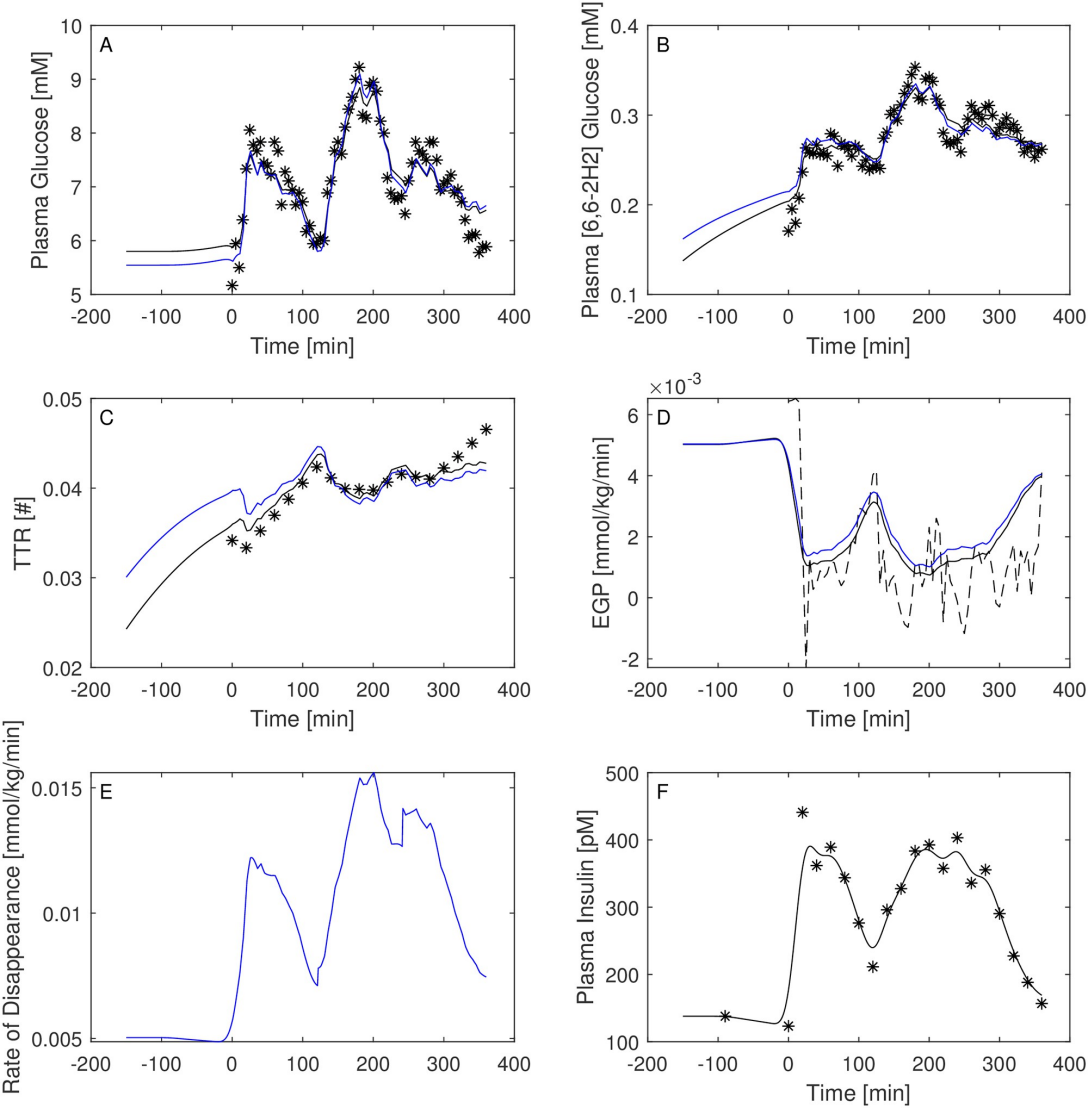
Predictions for Subject 6. Observed (asterisks) and predicted (line) variables over time (IGT subject). EGP: Endogenous Glucose Production; TTR: Tracer to Tracee ratio. Plasma glucose concentration (Panel A); Plasma [6, 6-2H2]glucose (Panel B); TTR (Panel C); EGP (Panel D), dashed line is the predicted EGP with the Steele model; Rate of disappearance (panel E); Plasma Insulin concentrations (Panel F). All the observed points refer to data measured on day 2. Plasma glucose concentrations (panel A) are derived from the glucose intra-venous infusion to match plasma glucose observations from the OGTTs of day 1. Black lines in panels A, B, C, D and E are model predictions from WLS estimation procedure; blue lines derive from the estimation approach which uses autocorrelated errors.

Figs 3–8 report the observations and the predictions of ‘total’ and ‘hot’ plasma glucose concentrations, as well as of TTR, along with the model predicted EGP (continuous lines) superimposed to the estimated EGP as derived by the Steele equation (dashed line). The last two panels report smoothed insulin concentrations and the estimated rate of glucose disappearance (given by the sum of insulin-dependent glucose uptake and by the zero-order brain uptake) for the six studied subjects under the one-compartment glucose model. While it is evident the good performance of the model in the studied subjects, it is especially important to consider the predictions of EGP. The mathematical formulation adopted produces predictions that can only assume positive values; furthermore, the paradoxical increase in EGP in the first minutes after the start of the experiment never occurs with the adopted model formulations. Panels from A to E show two different prediction lines, black and blue, derived from WLS parameter estimation procedure and from the approach taking into account autocorrelated observation errors. When the two procedures produce very similar results, the two curves in each panel are superimposed and only the blue line is evident.

**Fig 9:**
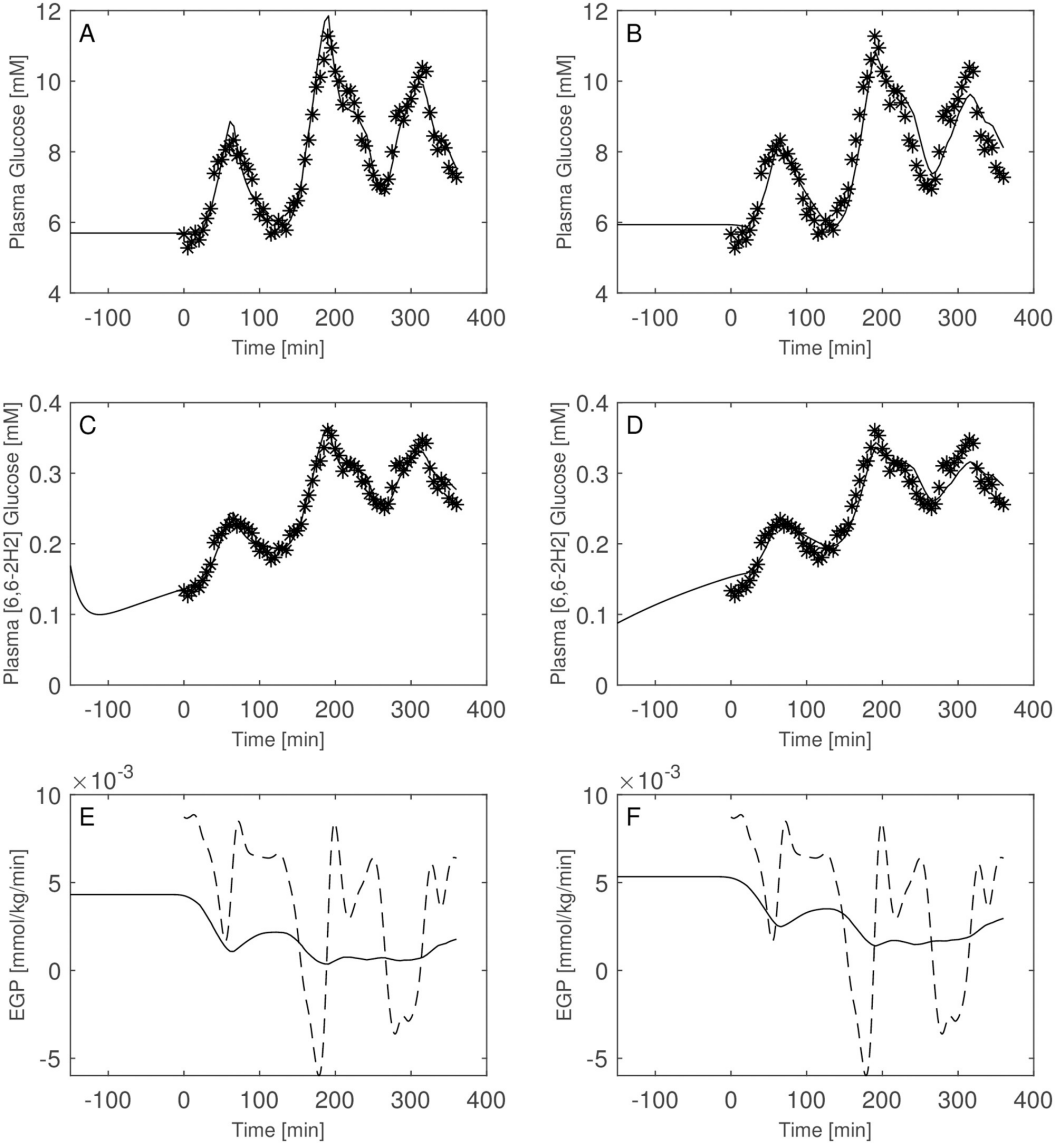
Model comparison for Subject 1. Observed (asterisks) and predicted (line) variables over time with glucose two-compartment model (Panels A, C, E) and one-compartments-glucose model (Panels B, D, F). Dashed line is the predicted EGP with the Steele model. All the observed points refer to data measured on day 2. Plasma glucose concentrations (panel A) are derived from the glucose intra-venous infusion to match plasma glucose observations from the OGTTs of day 1.

Of interest is the comparison between the performance of the two model formulations including or excluding the interstitial compartment. Figs 9 through 14 show the plasma glucose *G*_*P*_, the ‘hot’ glucose (*H*_*P*_) and TTR prediction together with their respective observations, as well as the estimated EGP for all patients with model A (not including the interstitial compartment, right panels) and model B (including the interstitial compartment, left panels). Table 4 reports the estimated distribution volumes (only plasmatic, *V*_*gpA*_, for the one-compartment glucose model and both plasmatic and interstitial, *V*_*gpB*_ and *V*_*giB*_, for the two-compartments glucose model) along with the transfer rate constants from the two compartments (plasma and interstitium) for model B as well as the loss function values, the Akaike (AIC) and BIC criteria obtained with the two formulations.

**Fig 10:**
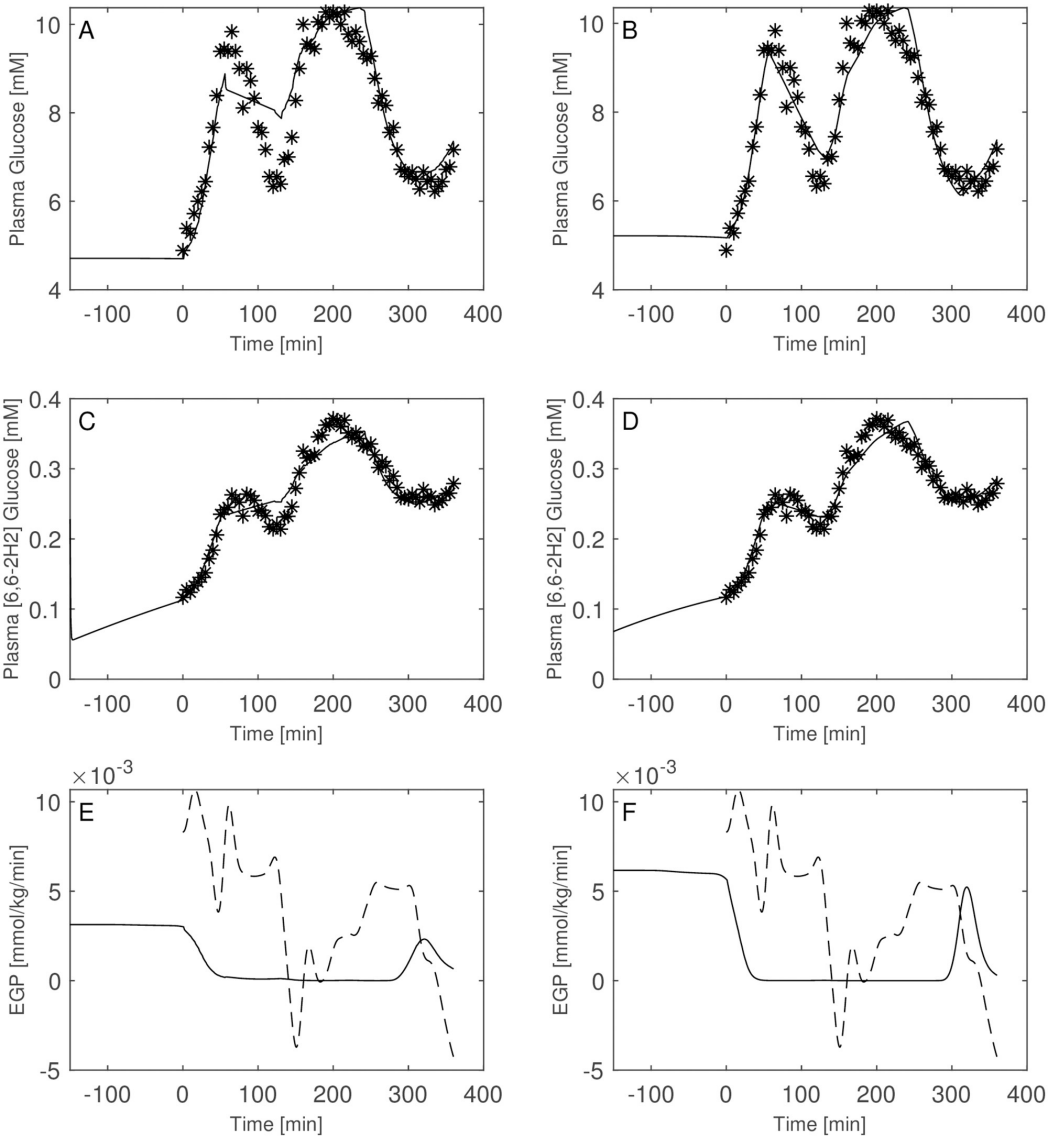
Model comparison for Subject 2. Observed (asterisks) and predicted (line) variables over time with glucose two-compartment model (Panels A, C, E) and one-compartments-glucose model (Panels B, D, F). Dashed line is the predicted EGP with the Steele model. All the observed points refer to data measured on day 2. Plasma glucose concentrations (panel A) are derived from the glucose intra-venous infusion to match plasma glucose observations from the OGTTs of day 1.

**Fig 11:**
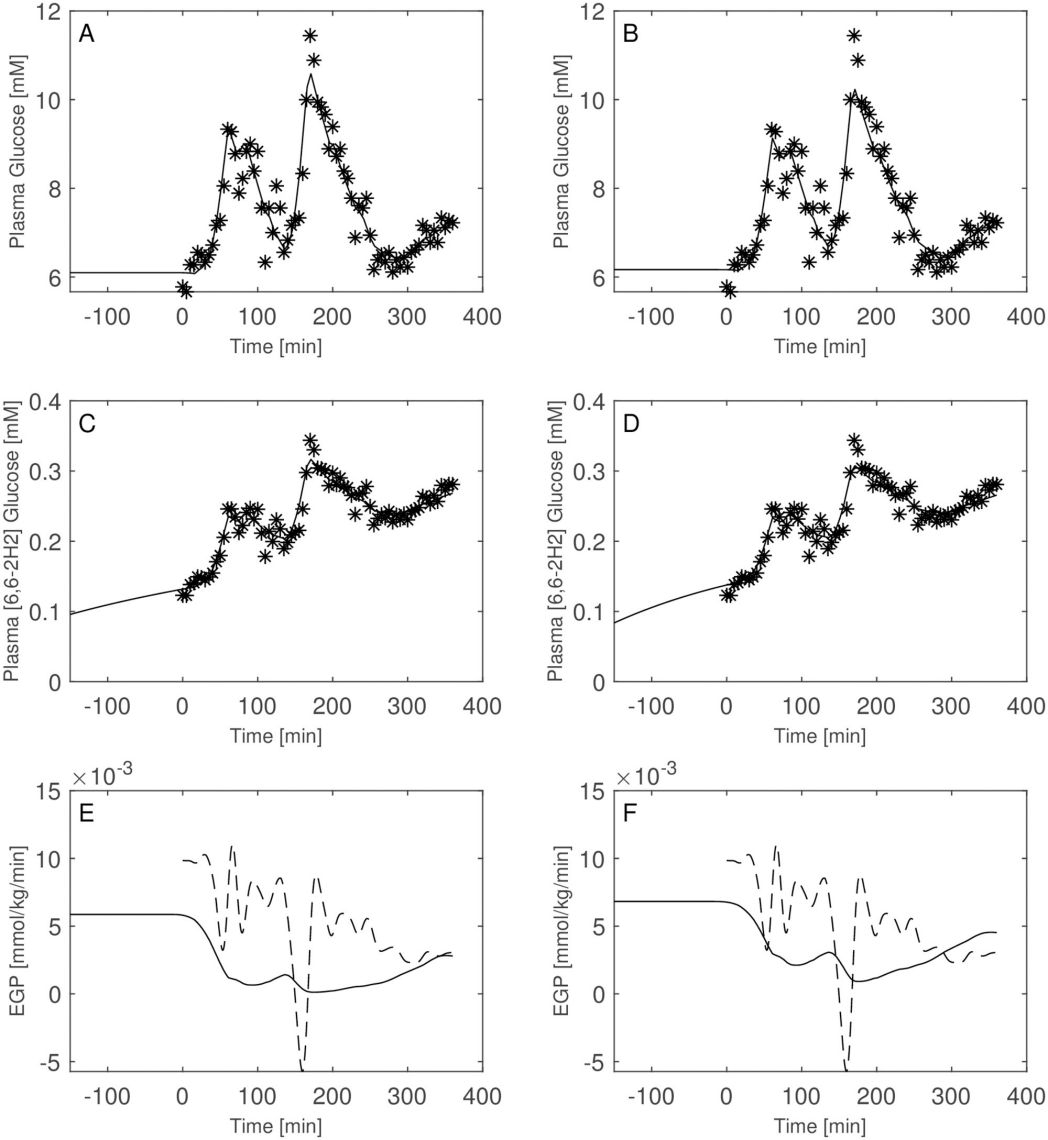
Model comparison for Subject 3. Observed (asterisks) and predicted (line) variables over time with glucose two-compartment model (Panels A, C, E) and one-compartments-glucose model (Panels B, D, F). Dashed line is the predicted EGP with the Steele model. All the observed points refer to data measured on day 2. Plasma glucose concentrations (panel A) are derived from the glucose intra-venous infusion to match plasma glucose observations from the OGTTs of day 1.

**Fig 12:**
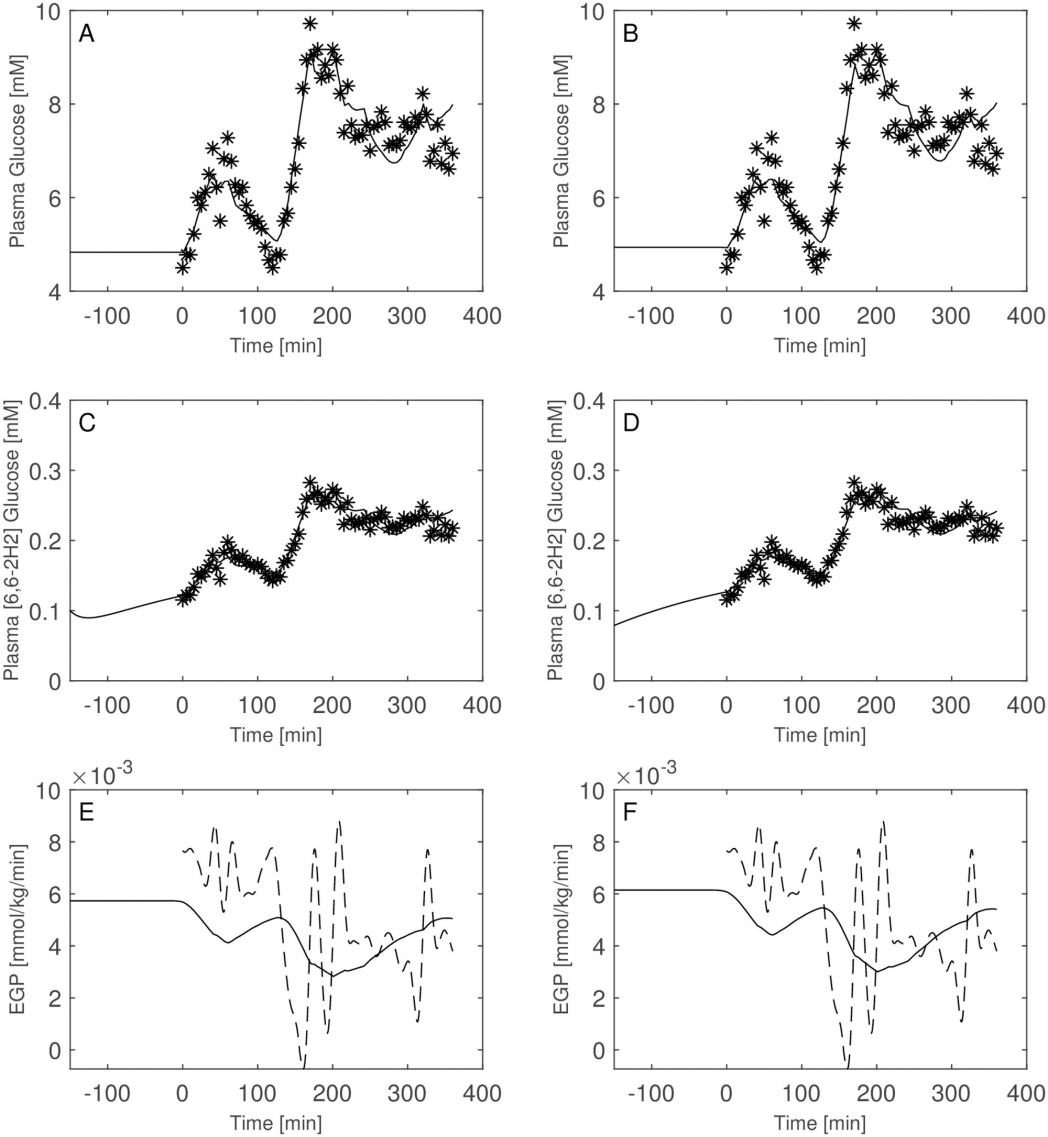
Model comparison for Subject 4. Observed (asterisks) and predicted (line) variables over time with glucose two-compartment model (Panels A, C, E) and one-compartments-glucose model (Panels B, D, F). Dashed line is the predicted EGP with the Steele model. All the observed points refer to data measured on day 2. Plasma glucose concentrations (panel A) are derived from the glucose intra-venous infusion to match plasma glucose observations from the OGTTs of day 1.

**Fig 13:**
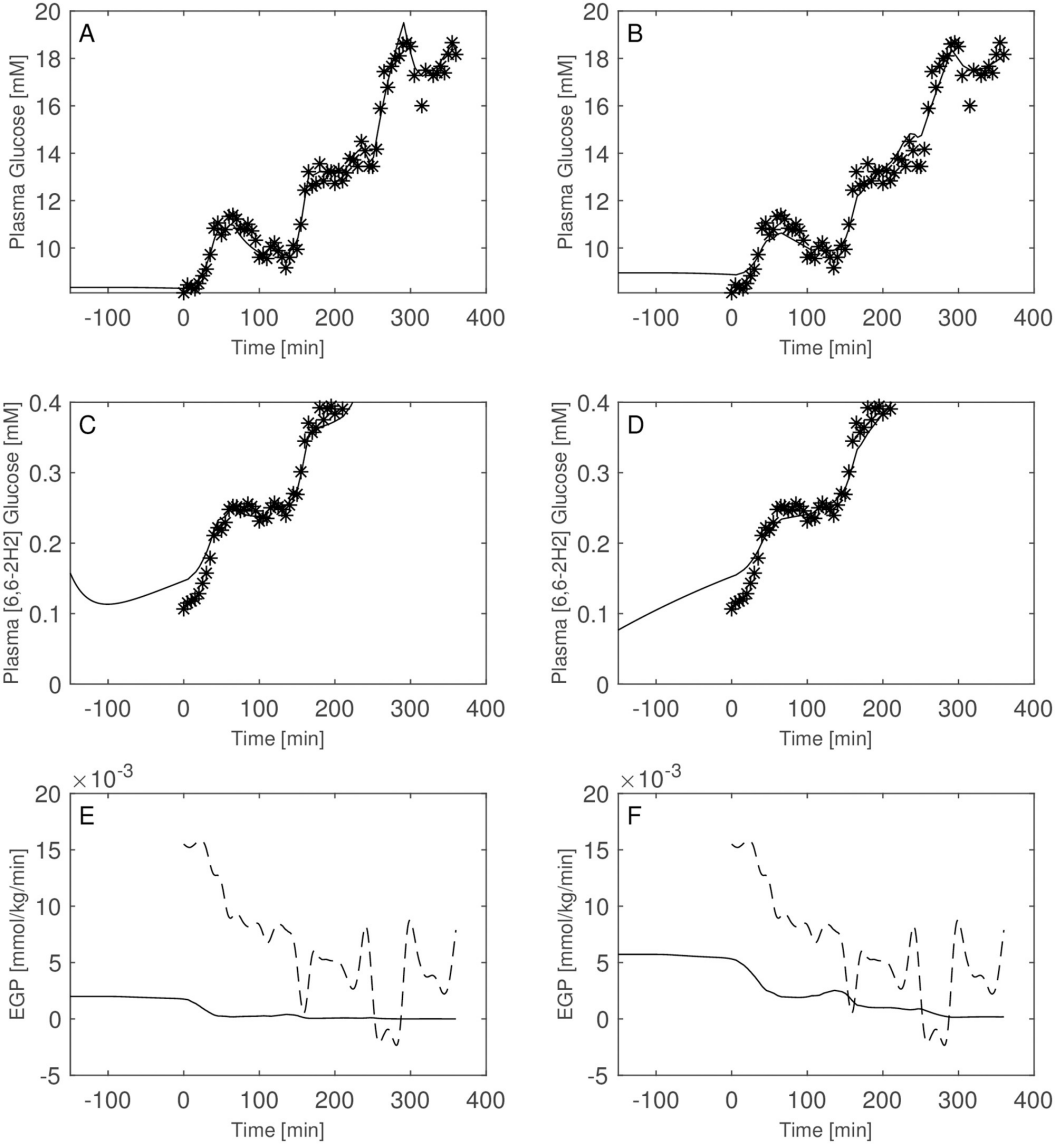
Model comparison for Subject 5. Observed (asterisks) and predicted (line) variables over time with glucose two-compartment model (Panels A, C, E) and one-compartments-glucose model (Panels B, D, F). Dashed line is the predicted EGP with the Steele model. All the observed points refer to data measured on day 2. Plasma glucose concentrations (panel A) are derived from the glucose intra-venous infusion to match plasma glucose observations from the OGTTs of day 1.

**Fig 14:**
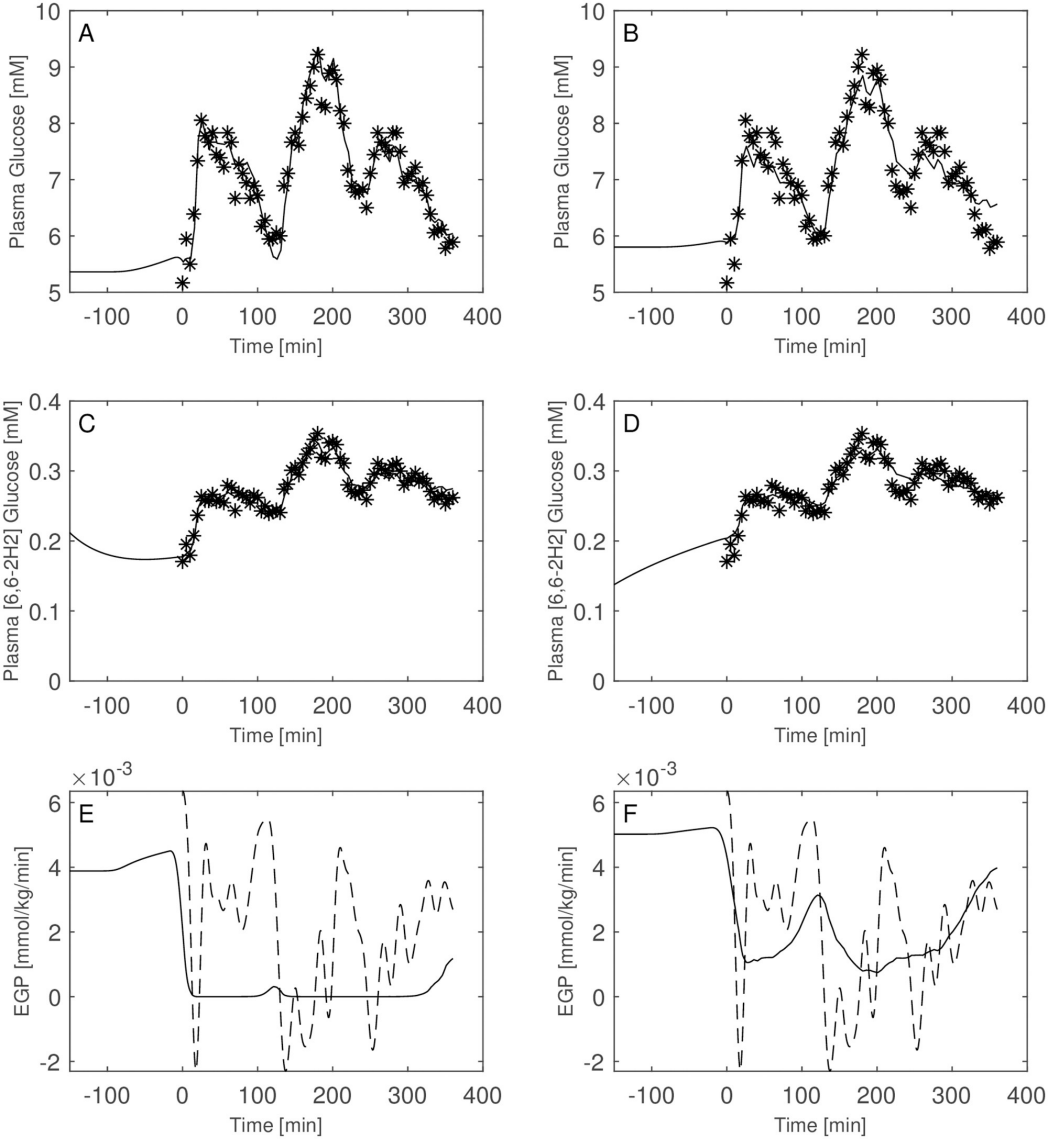
Model comparison for Subject 6. Observed (asterisks) and predicted (line) variables over time with glucose two-compartment model (Panels A, C, E) and one-compartments-glucose model (Panels B, D, F). Dashed line is the predicted EGP with the Steele model. All the observed points refer to data measured on day 2. Plasma glucose concentrations (panel A) are derived from the glucose intra-venous infusion to match plasma glucose observations from the OGTTs of day 1.

**Table 4:** Distribution volumes, loss, Akaike (AIC) and BIC criterium in the two model formulations: one-glucose compartment model (A) vs glucose two-compartments model (B). For Model B also the transfer rates between plasma and interstitium are reported.

Subject	*V*_*gpA*_	*Loss*_*A*_	*AIC*_*A*_	*BIC*_*A*_	*V*_*gpB*_	*V*_*giB*_	*k*_*igB*_	*k*_*giB*_	*Loss*_*B*_	*AIC*_*B*_	*BIC*_*B*_
1	0.251	0.786	110.02	127.92	0.130	0.197	0.039	0.026	0.278	111.97	135.84
2	0.324	0.395	117.4	135.3	0.096	0.320	0.643	0.194	0.731	122.86	146.72
3	0.263	0.459	93.60	111.50	0.230	0.148	0.003	0.004	0.421	97.45	121.32
4	0.279	0.635	59.94	77.84	0.220	0.087	0.015	0.038	0.597	63.78	87.65
5	0.287	0.711	217.16	234.89	0.140	0.274	0.024	0.012	0.539	220.46	244.11
6	0.160	0.368	113.29	131.19	0.104	0.233	0.008	0.003	0.180	116.52	140.39

**Fig 15:**
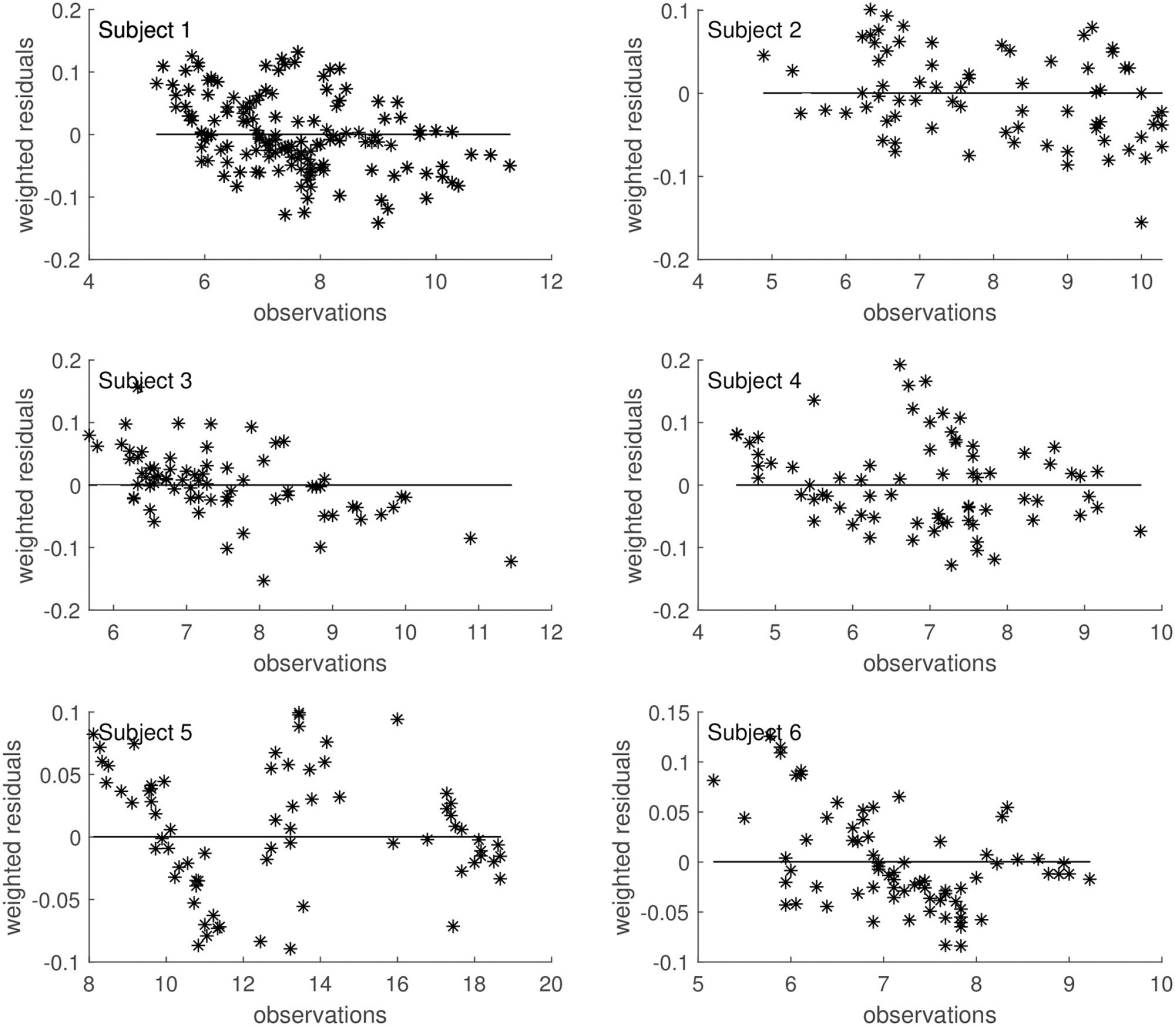
Weighted residuals. Scatter plot of weighted residuals towards observations under the hypothesis of correlated errors.

Both the AIC and BIC values privileged the simpler model (Table 4) and from the visual inspection (Figs 9–14) it is clear that in general little gain is obtained passing from the one-compartment glucose to the more complex two-compartment glucose model, except for Subject 2 for which the simpler model seems to perform better (Fig 10, panels A and C for the two-compartment glucose model and panels B and D for the one-compartment glucose model). Table 5 reports the estimates of the free parameters of the one-compartment glucose model under the WLS approach, whereas Table 6 reports the parameter estimates under the hypothesis of correlated errors. Fig 15 shows the scatter plot of weighted residuals towards observations. The average estimates and standard deviations derived from 14 are: *V*_*gp*_ = 0.246±0.036; *k*_*xGI*,1_ = 3.67×10^−05^±1.42×10^−05^; *k*_*xGI*,2_ = 2.37×10^−05^±9.42×10^−06^; *k*_*xGI*,3_ = 4.09×10^−05^±1.13×10^−05^; λ_*GI*_ = 0.0027±0.0013; *G*_*Pb*_ = 6.10±3.24. Values of the three indices of insulin sensitivity highlight for each studied subject a decreasing trend from the first to the second phase of the experimental procedure and an increasing trend from the second to the third phase, suggesting an intraday variability of the insulin sensitivity. On the contrary, Figs 4–7 clearly show the occurrence of the inappropriate initial EGP peak when using the Steele equation, which also produces in all subjects the typical (erroneous) negative EGP predictions. The EGP time courses obtained with the modelling approach proposed in the present work are in accordance with the observed and predicted glucose concentrations: after an initial fall from the basal condition (due to the start of the adjustable glucose infusion), the model-predicted EGP remains mostly constant or presents slight oscillations for the whole experimental procedure, increasing over the intervals where glucose concentrations are restored to their basal values. It is interesting, in this respect, to observe that in Fig 7 a persistent increase in glucose concentration corresponds to nearly zero liver glucose production.

**Table 5:** Parameter estimates of the one-glucose compartment model.

Subject	*V*_*gp*_	*k*_*xGI*,1_	*k*_*xGI*,2_	*k*_*xGI*,3_	λ_*GI*_	*G*_*Pb*_	*k*_*IG*,*max*_
1	0.251	5.31E-05	3.37E-05	5.33E-05	0.001	5.937	0.007
2	0.324	3.89E-05	1.64E-05	6.05E-05	0.018	5.213	0.491
3	0.263	3.42E-05	2.01E-05	2.50E-05	0.001	6.166	0.011
4	0.279	4.57E-05	3.30E-05	6.22E-05	0.0004	4.938	0.007
5	0.287	4.89E-06	3.49E-13	6.40E-06	0.001	8.951	0.106
6	0.160	1.97E-05	2.28E-05	2.78E-05	0.001	5.801	0.009
mean	0.260	3.27E-05	2.10E-05	3.92E-05	0.0037	6.17	0.105
SD	0.050	1.62E-05	1.13E-05	2.08E-05	0.0065	1.31	0.176

**Table 6:** Parameter estimates of the one-glucose compartment model with autocorrelated errors.

Subject	*V*_*gp*_	*k*_*xGI*,1_	*k*_*xGI*,2_	*k*_*xGI*,3_	λ_*GI*_	*G*_*Pb*_	*k*_*IG*,*max*_
1	0.251	5.31E-05	3.37E-05	5.33E-05	0.001	5.937	0.007
2	0.304	4.69E-05	2.02E-05	5.82E-05	0.0124	5.159	0.125
3	0.263	3.37E-05	2.01E-05	2.51E-05	0.0011	6.162	0.011
4	0.241	5.67E-05	4.04E-05	6.99E-05	0.0003	4.905	0.007
5	0.280	5.37E-06	3.68E-13	6.90E-06	0.001	8.906	0.079
6	0.136	2.43E-05	2.79E-05	3.22E-05	0.0006	5.545	0.008
mean	0.246	3.67E-05	2.37E-05	4.09E-05	0.0027	6.10	0.039
SD	0.053	1.79E-05	1.28E-05	2.15E-05	0.0043	1.32	0.046

## Discussion

Determination of glucose turnover, fasting and postprandial glucose concentrations is important for the assessment of possible impairment of glucose/insulin metabolism. Insulin resistance, both central and peripheral, plays an important role in the development of type 2 diabetes [44–46]. The liver is crucial for the maintenance of normal glucose homeostasis; it produces glucose during fasting and stores glucose as glycogen in the postprandial state: net hepatic glucose production derives from the sum of glucose fluxes determined by gluconeogenesis, glycogenolysis, glycogen synthesis, glycolysis and other pathways. In the postprandial state the liver helps to maintain normal glucose tolerance by absorbing glucose from plasma and storing it into glycogen, while hepatic glucose production (EGP) is suppressed.

Stable isotopes are currently used to measure glucose fluxes and for providing important information on hepatic and peripheral insulin resistance. Tracer techniques are commonly employed to estimate the rate of Appearance (RA) and the Rate of Disappearance (RD). In the post-prandial situation or during perturbation studies, estimation of RA and RD is rather complex due to gastric emptying, glucose absorption, appearance of ingested or infused glucose, variations of EGP and of glucose uptake.

The single-pool model by Steele [19–21] has been for a long time the most commonly employed approach for estimating RA, RD and EGP and is still widely employed in clinical investigations. However, the method suffers from at least two problems: on one hand it produces “paradoxical” increases in EGP immediately after glucose intake or after a meal, when, on the contrary, physiology would predict a compensatory fall in EGP. The Steele approach also leads to the computation of “negative” rates of EGP: it is to be kept in mind, in this context, that the EGP we refer to here is not a net glucose production balance by the liver, but a true hepatic glucose output, and that therefore negative EGP values are by definition not possible. Experimental designs with frequent sampling [28] have been employed to reduce these errors; Radziuk [29] suggested the use of a two-compartment model incorporating an accessible and a slowly exchanging nonaccessible compartment; other authors followed the dual-tracer approach, but the same problems were still encountered. Cobelli et al. [26] even proposed a more complicated method involving the use of three tracers for the estimation of the Rate of appearance (*Ra*_*meal*_), EGP and *R*_*D*_ after the ingestion of a meal together with variable IV tracer infusion rates so as to keep both all TTR’s constant. All of these methods are more expensive in terms of resources and human labor, and they do not satisfactorily solve the original problems anyway.

With the object of overcoming such problems, in this work a completely model-based approach is followed, using a simple compartmental model for glucose (either including or not including an explicit interstitial compartment). The proposed model is actually only one out of many possible models that can be used for the analysis of stable isotope or radioactive data, with the aim of estimating endogenous glucose production. What is here advocated is not the use of this particular model (even though this model appears to work well, at least for the present experimental application), but rather a shift to an altogether different approach: the simultaneous estimation of both ‘total’ and ‘hot’ glucose concentrations produces a more accurate assessment of tissue glucose uptake and of EGP, by representing them directly with simple but reasonable functions. By considering jointly the time courses of ‘hot’ and ‘total’ glucose concentrations, the glucose rate of disappearance can be estimated more precisely from the dynamics of ‘hot’ glucose, since RA is the only unknown in the equation. The RA of ‘cold’ glucose is then derived directly from the isotopic assumption, that ‘cold’ and ‘hot’ glucose are subject to the same metabolic processes.

To exemplify this approach, we applied it to an intra-venous experiment, where ‘cold’ glucose is infused at variable rates to reproduce a desired glycemic time-course: in this experiment, we have to deal with an intrinsically non-steady-state situation, since the glycemic profile we are attempting to reproduce is itself not constant.

We tested two different mathematical formulations: one including only one compartment for glucose distribution in the body (the plasma compartment) and one including an additional compartment for glucose distribution in the interstitial space. The one-compartment glucose model has only six free parameters to be identified from glucose concentration and TTR data, considering insulin as a forcing function. The introduction of the interstitial compartment with which plasma glucose reaches equilibrium through free diffusion (determined by the concentration gradient as formulated also in Rebrin and Steil [38]) introduces two more parameters to be estimated.

Judging on the basis of the present dataset and the currently estimated parameter values it is not clear whether the additional compartment is necessary. The results highlight a significant increase in fitting performance with the two-compartment glucose model only for patient 1 (see loss values in Table 4), with an equivalent EGP time course (see Fig 9). In two cases, in the face of an essentially equal performance of the two formulations (see for example Figs 11 and 12 for Subject 3 and 4 respectively), the estimated parameters suggest that the additional compartment may produce not plausible parameter values: the optimization procedure leads to smaller values for the interstitial distribution volume *V*_*gi*_ than expected, and produces smaller values for *V*_*gi*_ than for the plasmatic volume *V*_*gp*_. Moreover, for Subject 2 the simpler model produces a better data interpretation (as shown in Fig 4 and in Table 4). Given these considerations, along with the computed AIC and BIC values, the present observed data suggests the adoption of the more parsimonious formulation.

The mathematical formulation adopted for EGP avoids the occurrence of the two problems that emerge with the Steele formulation (Figs 4–7). The typical erroneous negative EGP predictions never happen, due to the intrinsically positive qualitative behaviour of the solutions for EGP. The paradoxical increase in EGP in the first minutes after the start of the experiment also does not occur. Our approach predicts nearly constant production rates after an initial decay immediately after the experimental glucose infusion, with few oscillations and for all subjects, with increasing endogenous glucose productions rates at the end of the experiment, when glucose concentrations tend to their basal values. This behaviour is in fact in excellent accord with the physiological understanding of the processes at play.

Other similar attempts of modelling the endogenous glucose production appeared in the literature [47, 48]. In the first work, the Krudys et.al proposed a compartmental model including three differential equations for the EGP production representation (amount of available glucose in the liver, endogenous glucose mass in the accessible compartment and endogenous glucose mass in the slowly equilibrating compartment), with a total of nine parameters plus other two parameters in the equation related to the remote insulin compartment. The model proposed by Krudys et.al therefore appears very expensive from the point of view of parameter estimation, especially in situations where the number of experimental observations is limited. In the work by Visentin et al., EGP is modelled by means of a total of two differential equations and two algebraic equations. The main equation describes the suppression of the EGP as proportional to the glucose rate of change, to glucose concentration and to the delayed insulin action. Two differential equations are used for the description of the dynamics of the delayed insulin action and one algebraic function is used for the contribution to the glucose rate of change, for a total of seven parameters, where one is determined from other model parameters (*EGP*_*b*_) and two other also appear in other model equations (*G*_*b*_ and *I*_*b*_), for a total of four additional parameters. Besides contemplating an extra parameter to be estimated, it is to be noted that, a-priori, the EGP from Eq (8) can assume negative values due to the fact that the negative terms appearing in Eq (8) are in principle unlimited. This is particularly true for the term proportional to the Glucose derivative which could be very large in correspondence of large glucose administration. A restricted region in the parameter space could be guarantee the positiveness of the solutions and should be investigated.

Our proposed formulation, instead, needs only one additional parameter (λ_*GI*_) to model the glucose contribution by the liver, since the parameter *K*_*IG*,*max*_ can be estimated by steady state conditions. In the present model EGP is represented as an input (an algebraic function) into the plasma glucose compartment, providing a more compact representation of the entire glucose/insulin system.

The estimated values obtained for the insulin sensitivity indices (parameter *k*_*xGI*,*j*_, *j* = 1, 2, 3) are of interest. In order to get a better fit of the model to the data, it was necessary to consider three different indices for the three phases of the experimental procedure, each quantifying the effect of insulin on glucose disposal in the corresponding phase. The trend of insulin sensitivity seems to be the same in all subjects: it decreases during the second phase and then increases corresponding to the third OGTT. These results are in agreement with the results of [49], according to which there is intra-day variability of insulin sensitivity. Even if the experiment lasted only 8.30 hours, less than a full day, and even if the variations over time in insulin sensitivity do deserve further ad hoc studies, the results obtained seem to be in line with what was hypothesized in [49]. In [49] the results showed that insulin sensitivity was on average lower at breakfast than at lunch and dinner, despite a great variability between subjects and despite the fact that evidence of a diurnal pattern was demonstrated in a study conducted only on subjects with type I diabetes. It is not possible to say with certainty that the increase observed in this series of experiments is the beginning of a trend leading to greater insulin sensitivity during the last hours of the day. However, the results obtained seem to give credence to this hypothesis.

## Conclusion

Although the model appears to fit fairly well with intravenous glucose data, future work will need to address a number of shortcomings. First of all, this approach will need to be adapted to the more complex setting of the estimation of glucose turnover in non-steady state conditions due to the ingestion of glucose. Also, insulin secretion and its effect on glucose disposal should be improved: in fact, while parameters can be reliably estimated under the assumption of autocorrelated error, the reason for such autocorrelation is still obscure. This may imply that the proposed model structure does not represent all necessary physiological mechanisms, or that these are represented in an overly simplistic form. Although improvements can be made, the strength of this model lies in the fact that it respects the physiological assumptions without producing artefacts, as otherwise happens with models which, while producing curves with an apparently better fit to the data, do so at the cost of producing implausible predictions, such as variables that take negative values. In the future it will be therefore necessary to devise a more complex model, which also includes a mathematical representation of the gastrointestinal tract, and because of this it will be necessary to estimate a larger number of parameters, while in any case ensuring that physiology is respected, for instance by exhibiting limited and positive solutions. In conclusion this work shows that appropriate, if simple, modelling of the whole infusion procedure, together with a coherent mathematical representation of the physiology, allows plausible, robust estimation of EGP without recourse to high-frequency sampling or complicated double or triple tracer administration. This approach prevents the artifactual occurrence of negative or spiking EGP predictions, as is instead produced by the Steele procedure.

## References

[1] Baley C (2005) Treating insulin resistance in type 2 diabetes with metformin and thiazolidinediones. Diabetes Obes Metab 7: 675–691. doi: 10.1111/j.1463-1326.2005.00497.x 16219011

[2] Mingrone G and Panunzi S and De Gaetano A and Guidone C and Iaconelli A and Leccesi L, et al. (2012) Bariatric surgery versus conventional medical therapy for type 2 diabetes. N Engl J Med 366: 1577–1585. doi: 10.1056/NEJMoa1200111 22449317

[3] Schauer P, Kashyap S, Wolski K, Brethauer S, Kirwan J, et al. (2012) Bariatric surgery versus intensive medical therapy in obese patients with diabetes. N Engl J Med 16: 1567–76. doi: 10.1056/NEJMoa1200225 22449319 PMC3372918

[4] Mingrone G, Panunzi S, De Gaetano A, Guidone C, Iaconelli A, et al. (2004) Bariatric-metabolic surgery versus conventional medical treatment in obese patients with type 2 diabetes: 5 year follow-up of an open-label, single-centre, randomised controlled trial. Lancet 386: 964–973. doi: 10.1016/S0140-6736(15)00075-6 26369473

[5] Schauer P, Bhatt D, Kirwan J, Wolski K, Aminian A, et al. (2017) Bariatric surgery versus intensive medical therapy for diabetes—5-year outcomes. N Engl J Med 376: 641–651. doi: 10.1056/NEJMoa1600869 28199805 PMC5451258

[6] Ikramuddin S, Korner J, Lee W, Thomas A, Connett J, et al. (2018) Lifestyle intervention and medical management with vs without roux-en-y gastric bypass and control of hemoglobin a1c, ldl cholesterol, and systolic blood pressure at 5 years in the diabetes surgery study. JAMA 319: 266–2778. doi: 10.1001/jama.2017.20813 29340678 PMC5833547

[7] Guidone C, Manco M, Valera-Mora E, Iaconelli A, Gniuli D, et al. (2006) Glucose-induced amplitude regulation of pulsatile insulin secretion from individual pancreatic islets. Diabetes 55: 2025–2031.16804072

[8] Gastaldelli A, Iaconelli A, Gaggini M, Magnone M, Veneziani A, et al. (2016) Short-term effects of laparoscopic adjustable gastric banding versus roux-en-y gastric bypass. Diabetes Care 39: 1925–1931. doi: 10.2337/dc15-2823 27573937

[9] Bojsen-Møller K, Dirksen C, Jørgensen N, Jacobsen S, Serup A, et al. (2014) Early enhancements of hepatic and later of peripheral insulin sensitivity combined with increased postprandial insulin secretion contribute to improved glycemic control after roux-en-y gastric bypass. Diabetes 63: 1725–1737. doi: 10.2337/db13-1307 24241533

[10] Castagneto Gissey L, Casella Mariolo J, Mingrone G (2018) Insulin granule trafficking in *β*-cells: mathematical model of glucose-induced insulin secretion. Peptides 100: 114–122. 29412812 10.1016/j.peptides.2017.12.010

[11] Ekberg K, Landau B, Wajngot A, Chandramouli V, Efendic S, et al. (1999) Contributions by kidney and liver to glucose production in the postabsorptive state and after 60 h of fasting. Diabetes 48: 292–298. doi: 10.2337/diabetes.48.2.292 10334304

[12] Mevorach M, Giacca A, Aharon Y, Hawkins M, Shamoon H, et al. (2013) Regulation of endogenous glucose production by glucose per se is impaired in type 2 diabetes mellitus. J Clin Invest 102: 744–753. 10.1172/JCI2720 PMC508937 9710443

[13] Pagliassotti M, Cherrington A (1992) Regulation of net hepatic glucose uptake in vivo. Annu Rev Physiol 54: 847–860. doi: 10.1146/annurev.ph.54.030192.004215 1562194

[14] Moore M, Coate K, Winnick J, An Z, AD C (2012) Treatment with the oral antidiabetic agent troglitazone improves beta-cell responses to glucose in subjects with impaired glucose tolerance. Adv Nutr 3: 286–294. 9239399 10.1172/JCI119562 PMC508219

[15] Cuezva J, Valcarce C, Chamorro M, Franco A, F M (1986) Alanine and lactate as gluconeogenic substrates during late gestation. FEBS Lett 194: 219–223. doi: 10.1016/0014-5793(86)80088-6 3940895

[16] Staehr P, Hother-Nielsen O, Landau B, Chandramouli V, Holst J, et al. (2003) Effects of free fatty acids per se on glucose production, gluconeogenesis, and glycogenolysis. Diabetes 52: 260–267. doi: 10.2337/diabetes.52.2.260 12540595

[17] Exton J, Park C (1968) Control of gluconeogenesis in liver. ii. effects of glucagon, catecholamines, and adenosine 3’,5’-monophosphate on gluconeogenesis in the perfused rat liver. J Biol Chem 243: 4189–4196. doi: 10.1016/S0021-9258(18)93242-4 5679958

[18] Lund A, Bagger J, Christensen M, Knop F, Vilsbøll T (2014) Glucagon and type 2 diabetes: the return of the alpha cell. Curr Diab Rep 14: 555. doi: 10.1007/s11892-014-0555-4 25344790

[19] Steele R, Wall J, De Bodo R, Altszuler N (1956) Measurement of size and turnover rate of body glucose pool by the isotope dilution method. Am J Physiol 187: 15–24. doi: 10.1152/ajplegacy.1956.187.1.15 13362583

[20] Steele R, Bishop J, Levine R (1959) Does a glucose load inhibit hepatic sugar output? c14 glucose studies in eviscerated dogs. Am J Physiol 197: 60–62. doi: 10.1152/ajplegacy.1959.197.1.60 13661389

[21] Steele R, Bjerknes C, Rathgeb I, Altszuler N (1998) Glucose uptake and production during the oral glucose tolerance test. Diabetes 17: 415–421. doi: 10.2337/diab.17.7.415 4875169

[22] Stefan N, Stumvoll M, Vozarova B, Weyer C, Funahashi T, Matsuzawa Y, et al. (2003) Plasma adiponectin and endogenous glucose production in humans. Diabetes Care 26: 3315–3319. doi: 10.2337/diacare.26.12.3315 14633820

[23] Koska J, Stefan N, Votruba SB, Smith SR, Krakoff J, Bunt JC (2008) Distribution of subcutaneous fat predicts insulin action in obesity in sex-specific manner. Obesity 16 (9). doi: 10.1038/oby.2008.292 18551127 PMC2692524

[24] Verbruggen SC, de Betue CT, Schierbeek H, Chacko S, van Adrichem LN, Verhoeven J, et al. (2011) Reducing glucose infusion safely prevents hyperglycemia in post-surgical children. Clinical Nutrition 30(6):786–792. doi: 10.1016/j.clnu.2011.05.011 21719165

[25] Hother-Nielsen O, Beck-Nielsen H (1990) On the determination of basal glucose production rate in patients with type 2 (non-insulin-dependent) diabetes mellitus using primed-continuous 3-3h-glucose infusion. Diabetologia 33: 603–610. 2257997 10.1007/BF00400204

[26] Rizza A, Toffolo G, Cobelli C (2016) Accurate measurement of postprandial glucose turnover: Why is it difficult and how can it be done (relatively) simply? Diabetes 65: 1133–1145. doi: 10.2337/db15-1166 27208180 PMC4839208

[27] Cobelli C FE Marl A (1987) Non-steady state: error analysis of Steele’s model and developments for glucose kinetics. Am J Physio 1252: E679–689. 10.1152/ajpendo.1987.252.5.E679 3578516

[28] Cobelli C, Toffolo G (1990) Constant specific activity input allows reconstruction of endogenous glucose concentration in non-steady state. Am J Physiol 258: E1037–E1040. 2193529 10.1152/ajpendo.1990.258.6.E1037

[29] Radziuk J (1976) An integral equation approach to measuring turnover in nonsteady compartmental and distributed systems. Bull Math Biol 38: 679–693. doi: 10.1007/BF02458642 990550

[30] Mingrone G and Panunzi S and De Gaetano A and Ahlin S and Spuntarelli V and Bondia-Pons I et al. (2020) Insulin sensitivity depends on the route of glucose administration. Diabetologia 63: 1382–1395. doi: 10.1007/s00125-020-05157-w 32385603 PMC7286868

[31] Cahill G (1970) Starvation in man. N Engl J Med 282(12): 668–675. 4915800 10.1056/NEJM197003192821209

[32] Felig P, Marliss E, Owen O, Jr C (1969) Blood glucose and gluconeogenesis in fasting man. Arch Intern Med 123: 293–298. doi: 10.1001/archinte.1969.00300130075011 4885676

[33] Gerich J (2010) Role of the kidney in normal glucose homeostasis and in the hyperglycaemia of diabetes mellitus: therapeutic implications. Diabet Med 27: 136–142. doi: 10.1111/j.1464-5491.2009.02894.x 20546255 PMC4232006

[34] Aussedat B, DupireAngel M, Gifford R, Klein J, Wilson G, et al. (2000) Interstitial glucose concentration and glycemia: implications for continuous subcutaneous glucose monitoring. Am J Physiol Endocrinol Metab 10: E716–E728. 10.1152/ajpendo.2000.278.4.E716 10751207

[35] Freeland A, Bonnecaze R (1999) Inference of blood glucose concentrations from subcutaneous glucose concentrations: applications to glucose biosensors. Ann Biomed Eng 27: 525–537. doi: 10.1114/1.196 10468237

[36] Steil G, Richey J, Kim J, Wi J, Rebrin K, et al. (1996) Extracellular glucose distribution is not altered by insulin: analysis of plasma and interstitial l-glucose kinetics. Am J Physiol 271: E855–E864. 8944672 10.1152/ajpendo.1996.271.5.E855

[37] Eddlestone G, Goncalves A, Bangham J (2014) Interstitium versus blood equilibrium in glucose concentration and its impact on subcutaneous continuous glucose monitoring systems. Eur Endocrinol 10: 36–42. 29872462 10.17925/EE.2014.10.01.36 PMC5983095

[38] Rebrin K, Steil G (2000) Can interstitial glucose assessment replace blood glucose measurements? Diabetes Technol Ther 2: 461–471. doi: 10.1089/15209150050194332 11467349

[39] Keenan D, Mastrototaro J, Voskanyan G, Steil G (2004) Delays in minimally invasive continuous glucose monitoring devices: a review of current technology. J Diabetes Sci Technol 3: 1207–1214. doi: 10.1177/193229680900300528 PMC2769894 20144438

[40] Vella A, Rizza RA (2009) Application of Isotopic Techniques Using Constant Specific Activity or Enrichment to the Study of Carbohydrate Metabolism. Diabetes 58(10): 2168–2174. 19794073 10.2337/db09-0318 PMC2750215

[41] Bergman R, Iyer M (2017) Indirect regulation of endogenous glucose production by insulin: The single gateway hypothesis revisited. Diabetes 66: 1742–1747. 28637826 10.2337/db16-1320

[42] Rebrin K, Steil G, Getty L, Bergman R (1995) Free fatty acid as a link in the regulation of hepatic glucose output by peripheral insulin. Science 44: 1038–1045. 7657026 10.2337/diab.44.9.1038

[43] Gastaldelli A, Coggan AR, Wolfe RR (1999) Assessment of methods for improving tracer estimation of non-steady-state rate of appearance. J Appl Physiol (1985) 87(5):1813–22 doi: 10.1152/jappl.1999.87.5.1813 10562626

[44] Reaven GM (1988) Role of insulin resistance in human disease. Diabetes 37: 1595–1607. doi: 10.2337/diab.37.12.1595 3056758

[45] Reaven G (2005) Insulin resistance, type 2 diabetes mellitus, and cardiovascular disease. The end of the beginning. Circulation 112: 3030–3032. doi: 10.1161/CIRCULATIONAHA.105.504670 16286599

[46] DeFronzo RA, Simonson D, Ferrannini E (1982) Hepatic and peripheral insulin resistance: A common feature of type 2 (non-insulin-dependent) and type 1 (insulin-dependent) diabetes mellitus. Diabetologia 23: 313–319. doi: 10.1007/BF00253736 6754515

[47] Krudys KM, Dodds MG, Nissen SM, Vicini P (2005) Integrated model of hepatic and peripheral glucose regulation for estimation of endogenous glucose production during the hot IVGTT Am J Physiol Endocrinol Metab 288(5):E1038–46 doi: 10.1152/ajpendo.00058.2004 15632105

[48] Visentin R, Dalla Man C, Basu R, Basu A, Rizza RA, Cobelli C (2015) Hepatic insulin sensitivity in healthy and prediabetic subjects: from a dual- to a single-tracer oral minimal model. Am J Physiol Endocrinol Metab 309(2):E161–7 doi: 10.1152/ajpendo.00358.2014 25991649 PMC4504934

[49] The UVA/Padova Type 1 Diabetes Simulator Goes From Single Meal to Single Day Journal of Diabetes Science and Technology 12(2): 273–281 10.1177/1932296818757747 PMC5851236 29451021

